# MORF2-mediated plastidial retrograde signaling is involved in stress response and skotomorphogenesis beyond RNA editing

**DOI:** 10.3389/fpls.2023.1146922

**Published:** 2023-03-28

**Authors:** Madhura M. Yapa, Paymon Doroodian, Zhenyu Gao, Peifeng Yu, Zhihua Hua

**Affiliations:** ^1^ Environmental and Plant Biology Department, Ohio University, Athens, OH, United States; ^2^ Interdisciplinary Program in Molecular and Cellular Biology, Ohio University, Athens, OH, United States; ^3^ State Key Laboratory of Rice Biology and Breeding, China National Rice Research Institute, Hangzhou, Zhejiang, China

**Keywords:** plastid, MORF2/RIP2, RNA editing, retrograde signaling, stress response, skotomorphogenesis, reactive oxygen species, inducible CRISPR Interference

## Abstract

Retrograde signaling modulates the expression of nuclear genome-encoded organelle proteins to adjust organelle function in response to environmental cues. MULTIPLE ORGANELLAR RNA EDITING FACTOR 2 (MORF2) was initially recognized as a plastidial RNA-editing factor but recently shown to interact with GUN1. Given the central role of GUN1 in chloroplast retrograde signaling and the unviable phenotype of *morf2* mutants that is inconsistent with many viable mutants involved in RNA editing, we hypothesized that MORF2 has functions either dosage dependent or beyond RNA editing. Using an inducible Clustered Interspaced Short Palindromic Repeat interference (iCRISPRi) approach, we were able to reduce the *MORF2* transcripts in a controlled manner. In addition to MORF2-dosage dependent RNA-editing errors, we discovered that reducing MORF2 by iCRISPRi stimulated the expression of stress responsive genes, triggered plastidial retrograde signaling, repressed ethylene signaling and skotomorphogenesis, and increased accumulation of hydrogen peroxide. These findings along with previous discoveries suggest that MORF2 is an effective regulator involved in plastidial metabolic pathways whose reduction can readily activate multiple retrograde signaling molecules possibly involving reactive oxygen species to adjust plant growth. In addition, our newly developed iCRISPRi approach provided a novel genetic tool for quantitative reverse genetics studies on hub genes in plants.

## Introduction

Chloroplasts not only function as a powerhouse converting light energy into sugar through photosynthesis, but also compartmentalize multiple metabolic pathways for the biosynthesis of amino acids, fatty acids, and phytohormones (Chan et al., 2016). Given their vital importance, intensive studies have been focused on the function and development of chloroplasts (Jarvis and Lopez-Juez, 2013). One central topic is about the coordination between nuclear gene expression and the physiological and developmental status of chloroplasts, named anterograde (nucleus to chloroplasts) and retrograde (chloroplasts to nucleus) signaling (Chan et al., 2016). Because >95% of the chloroplast proteins are encoded by nuclear genes with the remainder encoded by the chloroplast genome, the expression of these two sets of proteins needs to be promptly controlled to maintain the stoichiometry of protein complexes assembled in the chloroplasts (Woodson and Chory, 2008). The activity of these complexes is sensitive to environmental cues such as light quality and/or intensity (Woodson and Chory, 2008; Chan et al., 2016). Since not all absorbed photons from photosystems are fixed, excess ones often alter the reduction/oxidation (redox) state of the photosynthetic electron transfer chain and induce oxidative damage (Bassi and Dall'osto, 2021). Additional stresses can further disrupt the balance of redox and metabolites in chloroplasts and promote the formation of reactive oxygen species (ROS) (Chan et al., 2016; Li and Kim, 2022). Increasing ROS and metabolic changes generate multiple signals, so called retrograde signaling (RS), to orchestrate vast expression changes of nuclear genes involved not only in chloroplast photoprotection but also in other intracellular functions (Chan et al., 2016; Bassi and Dall'osto, 2021; Dopp et al., 2021).

Emerging evidence has demonstrated a central role of chloroplasts/plastids in stress defense through RS. For example, the lincomycin-induced RS has been discovered to suppress and activate phytochrome and ethylene pathways, respectively, resulting in inhibition of photomorphogenesis of seedlings growing under a moderate light condition. The discoveries from these studies suggest a photoprotection role of RS during seedling de-etiolation (Martin et al., 2016; Gommers et al., 2021; Veciana et al., 2022). Given the presence of multiple RS molecules, it is plausible that some may have no impact on plant photomorphogenesis, such as that resulting from the arrested albino plastids of *pap7-1*, a mutant that lacks plastid-encoded RNA polymerase-associated proteins (Grubler et al., 2017). In addition to photomorphogenesis, the RS molecule, methylerythritol cyclodiphosphate (MEcPP), induces an array of nuclear genes responsible for general stress response (Walley et al., 2015; Benn et al., 2016).

While RS regulates the expression of nuclear-encoded chloroplast proteins, chloroplast genome-encoded proteins are under an organelle-specific posttranscriptional regulation, called RNA editing (Small et al., 2020). Although a multigene protein family composed of pentatricopeptide repeat (PPR) proteins has been demonstrated for specific recognition and/or cytidine deamination of RNA-editing sites, the biochemical roles of many other PPR-interacting factors are unknown (Takenaka, 2014; Small et al., 2020). Among these factors is a small family of nuclear genome-encoded proteins, termed MULTIPLE ORGANELLAR RNA EDITING FACTORs (MORFs). Mutant studies and protein-protein interaction analyses suggest that they are involved in a devoted RNA editosome (Takenaka et al., 2012). Although RNA editing was first documented 30 years ago, its biological influence is yet not clear because several Arabidopsis mutants showing RNA-editing errors both in mitochondria and chloroplasts have no or only mild growth defects (Takenaka et al., 2010; Sun et al., 2013). However, the null mutant allele, *morf2-1*, is albino with errors/deficiencies in multiple chloroplast RNA editing sites. Further analyses identified physical interactions of MORF2 with selected PPR proteins (Takenaka et al., 2012). Despite these, the detailed biochemical function of MORF2 in RNA editing is yet not clear. Intriguingly, MORF2 was recently shown to interact with GENOMES UNCOUPLED 1 (GUN1), one of the central players in RS. Transcriptomic analyses discovered a *gun* molecular phenotype in *MORF2* overexpression plants, suggesting its negative function in RS regulation. Moreover, compared to wild type (WT), differential RNA-editing profiles were observed in both *gun1* null mutant and the *MORF2* overexpression lines when treated with the bleaching herbicide norflurazon (NF) (Zhao et al., 2019). Although this discovery suggests a possible interplay between RNA editing and RS, it remains elusive whether and how the observed RNA-editing changes result from RS or *vice versa* (Larkin, 2019; Zhao et al., 2019).

Delineating MORF2-mediated early interaction between RS and RNA editing would further our understanding about the biological function of RNA editing, explore the formation of RNA editosome, and categorize differential RS pathways in plant stress response. Because all up-to-date genetic studies in RS and RNA editing involve either strong pharmacological treatments or knockout mutants, which result in severe or no phenotypes, the early signaling in both pathways remains unknown. In this work, we applied a novel inducible Clustered Regularly Interspaced Short Palindromic Repeat interference (iCRISPRi) approach to quantitatively monitor early transcriptome and RNA-editing changes upon the gradient reduction of MORF2. Further physiological and expression analyses, in combination with the structure feature and a recent finding of a holdase-like chaperon activity of MORF2 (Yuan et al., 2022), collectively suggest that MORF2 is a stress effector in addition to its previously discovered role in RNA editing.

## Materials and methods

### Plant materials and growth conditions

**Figure 1:**
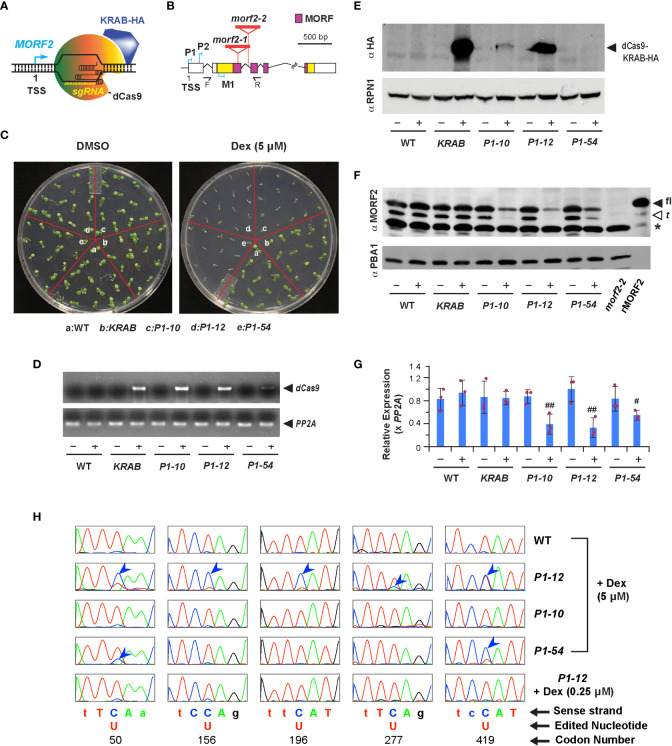
Downregulation of MORF2 by iCRISPRi inhibits plant growth prior to influencing RNA editing in chloroplasts. **(A)** A diagram showing the binding of dCas9-KRAB and sgRNA to the *MORF2* locus. TSS: transcription start site. **(B)** Schematic representation of the *MORF2* locus. Folded lines indicate introns, and the colored and white boxes represent coding and untranslated regions, respectively. Regions encoding the MORF domain are pink colored. Red triangles locate the T-DNA insertion sites for the *morf2-1* and *morf2-2* alleles. P1, P2, and M1 indicate the position of three protospacers. F and R represent the site of forward and reverse primers, respectively, used in combination with LBa1 (a left border primer of the T-DNA) for confirming the homozygous *morf2-2* allele by PCR. Primer sequences are listed in **Table S1**. **(C)** Growth phenotypes of 7-d-old seedlings grown under LD on 1/2 MS media supplemented with 0.1% DMSO (DMSO hereafter) or 0.1% DMSO plus 5 μM Dex (5 μM Dex hereafter). **(D)** sqRT-PCR identifies an induced expression of *dCas9* only in the indicated transgenic plants treated with 5 μM Dex as shown in **(C)**. – and + indicate DMSO and 5 μM Dex treatments, respectively, herein as well as in **(E-G)**. **(E)** Immunoblot analysis showing the detection of dCas9-KRAB-HA fusion in *KRAB*, *P1-10*, and *P1-12* seedlings treated with 5 μM Dex as shown in **(C)**. A proteasome subunit, RPN1, was used as a control indicating nearly equal protein loading. **(F)** Dex treatment reduced protein products of MORF2 in three *P1* transformants but not in *KRAB*. Protein extracts from 7-d-old *morf2-2* seedlings and *E. coli* cells expressing recombinant MORF2 (rMORF2) were used as negative and positive controls showing the absence and presence of MORF2, respectively. The full-length (fl) and truncated (t; putatively processed by peptidases) MORF2 are indicated by the closed and open arrowheads, respectively. The asterisk identifies an unknown cross-reacting species. A proteasome subunit, PBA1, was used as a control indicating nearly equal protein loading. **(G)** qPCR analysis of *MORF2* transcripts in 7-d-old seedlings treated with or without 5 μM Dex as shown in **(C)**. Relative expression of *MORF2* is normalized to one of the three WT biological replicates treated with DMSO. *PP2A* shown in this figure and in **Figures 2E**, **3E**, **7** was used as reference standards. Bars represent mean ( ± SD) from three biological replicates, each with three technical replicates. The maroon dots indicate three data points of replicates in each bar. # and ## indicate statistically significant differences between the two treatments within the same genotype (Student’s *t*-test, *P* < 0.05 and 0.01, respectively). **(H)** Comparison of sequencing chromatograms containing five indicated editing sites in the transcripts of *ndhB* gene obtained from 7-d-old light grown seedlings as indicated. Blue arrowheads point to the cytidine sites with lower editing efficiencies to uridine compared to WT. Genetic codons containing an editing site are capitalized and numbered.

Unless otherwise noted, the Arabidopsis reference accession Col-0 was used as the WT control. The mutants, *morf2-2* (stock number: SALK_094930) and *ctr1-1* (stock number: CS8057), were obtained from the Arabidopsis Biological Resource Center (ABRC) at the Ohio State University. Locations of the T-DNA insertion in *morf2-2* was confirmed by genomic Polymerase Chain Reaction (PCR) analysis using the primers indicated in **Figure 1B**. All oligonucleotide primers used in this study are listed in **Table S1**.

Seeds harvested at the same time from plants grown under an identical environmental condition were used for growth assay. Dried seeds were vapor-phase surface sterilized for 3 to 5 h in the presence of 100 mL NaOCl (bleach) supplemented with 3 mL HCl. For seedlings grown under a long day (LD) photoperiod (16-h white light (120 μmol m^-2^ s^-1^)/8-h darkness at 21**°**C and 19**°**C, respectively), seeds were stratified in water at 4°C under darkness for three days before germination on half-strength Murashige-Skoog (1/2 MS; Caisson Labs) medium plus 1% (w/v) sucrose. For dark or dim light grown seedlings, seeds were briefly hydrated in water and plated on 1/2 MS without sucrose for stratification at 4°C under darkness for three days before germination. Dark grown seedlings were germinated in sealed two layers of aluminum foil wrap for three days with the same temperature condition as LD upon a 6-h germination initiation under white light (120 μmol m^-2^ s^-1^). Dim light grown seedlings were directly germinated and treated under continuous white light (1.5 μmol m^-2^ s^-1^) and temperature (23°C) for three days. Different concentrations of dexamethasone (Dex; MilliporeSigma), 1-Aminocyclopropane-1-carboxylate (10 μM ACC; MP Biomedicals), sliver nitrate (5 μM AgNO3; MilliporeSigma), lincomycin (Linc, 0.5 mM; MilliporeSigma), norflurazon (NF, 5 μM; MilliporeSigma), hygromycin B (30 mg/L; Calbiochem), and phosphinothricin (15 mg/L; Gold Biotechnology) were filter sterilized and added into the 1/2 MS medium after autoclave (121°C, 30min).

To prepare plants for seed propagation and transformation, 7-d-old seedlings grown on 1/2 MS under LD were planted on mixed soil containing 1/3 vermiculite, 1/3 peat moss, and 1/3 compost soil and grown under LD. Adult plants were fertilized once before bolting with 0.1% (w/v) 20-20-20 (Miracle-Gro), Calcium Nitrate (0.17 mM) and Magnesium Sulfate (0.33 mM).

### Plasmid construction and plant transformation

The coding sequence for the fusion protein Flag-dCas9-KRAB-HA was PCR amplified from pHSN6I01 (addgene, Plasmid #50587) and cloned into the XhoI-SpeI sites of pTA7002 binary transformation vector to yield pTA7002-*Dex_p_:KRAB* (Aoyama and Chua, 1997). Upon ligation, an AscI site was introduced in front of SpeI site, between which an expression cassette for *P1*, *P2*, or *M1* synthetic single-guide RNA (*sgRNA*) gene was inserted to generate a dual expression construct, pTA7002-*Dex_p_:KRAB U6-26_p_:P1, P2*, and *M1*, respectively. Each *sgRNA* expression cassette containing *U6-26_p_*, *sgRNA* gene and *U6-26_t_* was constructed by overlapping PCR using pCBC-DT1T2 (addgene, Plasmid # 50590) as a template. To construct *Yellow Fluorescent Protein* (*YFP*) tagged *35S:MORF2-YFP* overexpression construct, the coding sequence of *YFP* was first PCR amplified from pEarleyGate101 (ABRC, #CD3-683) and cloned into pFGC5941 binary transformation vector between BamHI and XbaI sites. Upon construction, a 39-nucleotide DNA fragment encoding for a 13-amino acid linker (GSAGGGGGGGGGG) was introduced in-frame at the 5’-end of *YFP*. The resulting construct was further introduced with a coding sequence for HA or MORF2 in the AscI-BamHI sites in fusion with *YFP* to yield pFGC5941-*35S:HA-YFP* or pFGC5941-*35S:MORF2-YFP*, respectively.

All constructions were sequence-confirmed and introduced into the *Agrobacterium tumefaciens* strain GV3101, which was used to transform Col-0 using the floral dip method (Bent, 2006). Plants containing a single transgenic insertion were identified based on a 3:1 (resistant/sensitive) segregation ratio of T2 plants grown on 1/2 MS medium containing 30 mg/L hygromycin B for plants transformed with transgenes cloned in pTA7002 vector, or 15 mg/L phosphinothricin for plants transformed with transgenes cloned in pFGC5941 vector. Unless otherwise noted, all the experiments were performed using T4 or T5 progeny of homozygous transgenic plants carrying one transgenic insertion.

### RNA isolation and gene expression analysis

Total RNA was extracted from 10-50 mg seedlings using NucleoSpin RNA Plus kit (Macherey-Nagel) in accordance with the manufacturer’s protocol. Three independent biological samples were prepared for each assay. From each sample, 1.5 μg of total RNA was treated with DNase I (Thermo Fisher Scientific) for 15 min at room temperature prior to be converted into cDNA using the SuperScript III first-strand synthesis system (Thermo Fisher Scientific) following the manufacturer’s instructions. The resulting cDNA was then used as a template for either semi-quantitative Reverse-Transcription PCR (sqRT-PCR) or real-time quantitative (qPCR) analyses with primers listed in **Table S1**.

qPCR was performed on a Bio-Rad CFX Connect real-time PCR detection system using PowerUp™ SYBR™ Green Master Mix (Thermo Fisher Scientific) with three technical replicates for each of three biological samples. In a 10 μL qPCR reaction, 4 μL of cDNA (diluted 1/30 following first-strand synthesis) was amplified with 5 μL PowerUp™ SYBR™ Green Master Mix, and 0.5 μL each of 10 μM forward and reverse primers. Reactions were run in a cycling program consisting of 95°C for 5 min, 45 cycles of 95°C for 10 sec, 60°C for 10 sec, and 72°C for 30 sec, and followed by a melting curve program and cooling at 40°C for 2 min. Fluorescence data were collected at the end of each 72°C extension step, and continuously during the melting curve program. Relative gene expression was calculated according to the 2-ΔΔCt method with *SERINE/THREONINE PROTEIN PHOSPHATASE 2A* (*PP2A*) used as an internal control.

For transcriptome sequencing, two replicates of total RNA were isolated using the same method as described above from 8-d-old *KRAB* and *P1-12* seedlings grown vertically on 1/2 MS media supplemented with or without different concentrations of Dex. For each sample, 1 μg RNA was used for pair-end 150-mer deep sequencing on a NovaSeq 6000 platform (Novogene). Adapter and low-quality sequences were removed from the raw FASTQ sequencing data (available at the Gene Expression Omnibus under accession number GSE213405) using Trimmomatic [phred=33, minimum length=36; (Bolger et al., 2014)]. The resulting valid sequences were aligned to the *A. thaliana* Col-0 reference genome (TAIR10; www.arabidopsis.org) using TOPHAT2 (Kim et al., 2013) to identify accepted hits of each locus. HTSeq was then used to calculate an absolute expression level (counts) of each locus based on the accepted hits (Anders et al., 2015).

**Figure 2:**
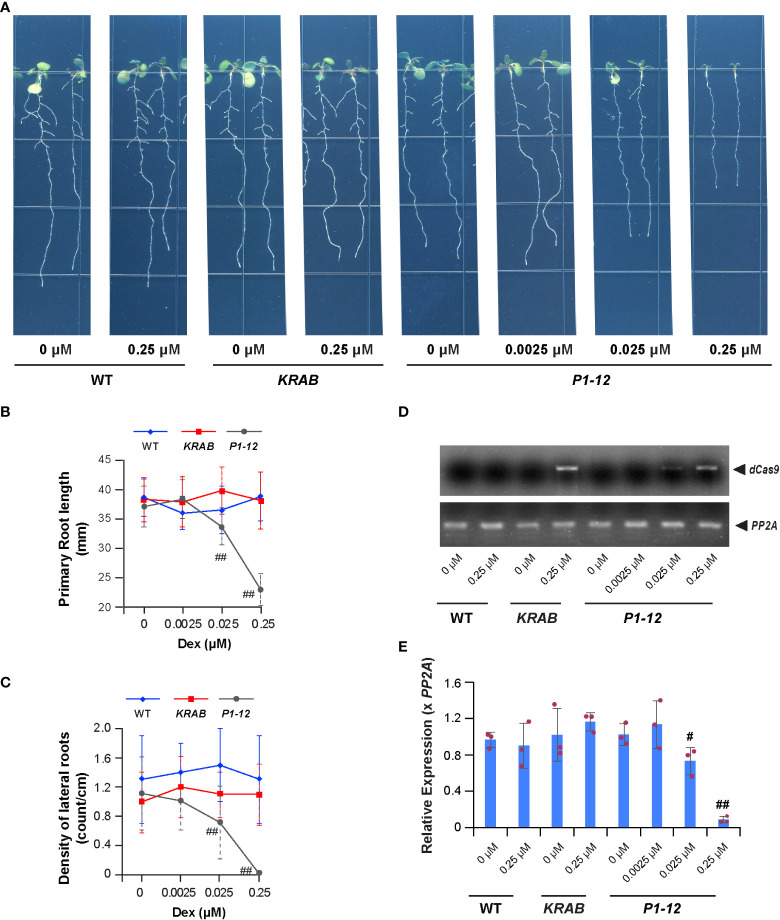
A MORF2 dosage-dependent growth inhibition. **(A)** Representative images showing seedlings from indicated genotypes grown vertically for 8 d under LD on 1/2 MS media supplemented with different concentrations of Dex. **(B, C)** Dex treatment inhibits primary **(B)** and lateral **(C)** root growth of *P1-12* but not *KRAB*. The seedlings shown as in **(A)** were scanned and measured for primary root length using NeuronJ plugin in ImageJ. Visual lateral root number was counted manually. The density of lateral roots was normalized by the primary root length. **(D)** sqRT-PCR identifies the expression of *dCas9-KRAB* gene in seedlings of *KRAB* (+0.25 μM Dex) and *P1-12* (+0.025/0.25 μM Dex). *PP2A* amplicons were shown to indicate a similar input of cDNA templates among different samples. **(E)** Dex treatment inhibits *MORF2* expression in *P1-12* at a concentration as low as 0.025 μM. Relative expression (mean ± SD) was normalized to *PP2A* from three biological replicates, each with three technical replicates. All data points were further normalized to one of the three biological replicates of WT seedlings grown under the DMSO treatment. The data points of replicates in each bar are indicated with maroon dots. *P* values were calculated using Student’s *t*-test. # and ##: *P* (< *P1-12* (+DMSO)) < 0.05 and 0.01, respectively.

To maximize cross experimental comparisons, we applied two standards for determining an expressed gene. For Significantly Differentially Expressed Gene (SDEG) analysis between *KRAB* and *P1-12* seedlings shown in **Figure 2**, raw expression data (counts) from 10 transcriptomes with two from each of *P1-12* (+DMSO), *P1-12* (+0.025 μM), *P1-12* (+0.25 μM), *KRAB* (+DMSO), and *KRAB* (+0.25 μM) were normalized to counts per million (CPM). A gene was considered as expressed if its CPM was above 2 in at least nine transcriptomes compared. For cross experimental comparisons, raw counts were normalized among 12 transcriptomes with two from each of *P1-12* (+DMSO), *P1-12* (+0.025 μM), Col6-3 (LS for 1/2 Linsmaier and Skoog medium), Col6-3 (+NF), *gun1-9* (+NF), and *morf2-2* (GM for Gamborg’s B5 basal medium). A gene was retained as expressed if it had a greater than 0.5 CPM in at least 11 samples. Similarly in the second cross experimental comparison for studying ethylene-regulated genes, we normalized the raw counts of four transcriptomes from *P1-12* (+DMSO) and *P1-12* (+0.25 μM) and considered a gene as expressed if it was expressed above 0.5 CPM in at least three samples. Normalized transcriptomes of expressed genes were further analyzed to calculate relative expression (Log_2_FC, FC for fold changes) and SDEGs ([Log_2_FC] ≥ 1; FDR < 0.01%) using edgeR (Robinson et al., 2010).

GO enrichment analysis was performed using a classical enrichment analysis embedded in the topGO package (Alexa et al., 2006). The assignment of GO functional categories was based on the TAIR GO categories from aspect “biological process” (ATH_GO_GOSLIM_20150408.txt) (Berardini et al., 2004). All GO terms at level 2 with *P* < 1e-10 (Fisher’s exact test) were considered to be overrepresented within each group of SDEGs. The list of SDEGs in GO:0009723 term was resolved using the “sigGenes” and “genesInTerm” functions in the topGO package (Alexa et al., 2006). Clustering analysis was performed using Heatmap.2 in R (http://www.r-project.org) based on row Z-scores of each expressed gene calculated by normalized CPM or relative expression.

### RNA editing efficiency

Total RNA was extracted from 7-d-old LD-grown seedlings treated with different concentrations of Dex and converted to cDNA as described above. PCR fragments of *ndhB* were specifically amplified from the cDNA and sequenced using the primers listed in **Table S1**. The RNA editing efficiency at each site was measured by the proportion of sequenced thymine chromatogram area to that of cytosine at the editing site analyzed.

Transcriptome-wide analysis of chloroplast RNA-editing efficiency is performed using the RNA sequencing data obtained from 8-d-old *KRAB* and *P1-12* seedlings upon Dex or DMSO treatments. The accepted reads of each locus obtained above in transcriptome analysis were sorted according to their positions in the Arabidopsis genome and saved as an aligned Binary Alignment Map (BAM) file. The resulting BAM file was further used to generate an index file using SAMtools (Li et al., 2009). Both position-sorted BAM and index files were loaded into the Integrative Genomics Viewer (IGV) program (Robinson et al., 2023) for manually examining the counts of thymine and cytosine nucleotides present in reads aligned with a specific editing site to digitally represent its editing efficiency. For each editing site, the counts of two biological replicates of the same genotype were combined and used to determine its statistically significant change of editing efficiency upon Dex treatment using Fisher’s exact test. All 33 known chloroplast RNA-editing sites as described in Takenaka et al. (Takenaka et al., 2012) were examined.

### Immunoblotting analysis

7-d-old LD-grown seedlings treated with or without 5 μM Dex were harvested, pulverized in liquid nitrogen, and immediately used for total protein extraction in 2 x SDS-PAGE sample buffer at 95°C for 5 min. Upon being resolved in SDS-PAGE, proteins were electrophoretically transferred onto polyvinylidene difluoride membranes (Immobilon-P; Millipore Sigma) for immunoblotting analysis.

MORF2 antibodies were raised against a MORF2-6His fusion in rabbits (Envigo Bioproducts). The antigenicity of MORF2 antibodies were verified by the absence and presence of protein bands with the expected size of full-length MORF2 (flMORF2) in *morf2-2* and a tag-free recombinant MORF2 (rMORF2), respectively. rMORF2 was produced in BL21 *E. coli* cells transformed with pET28-MORF2, in which the CDS of *MORF2* was inserted in the NcoI-EcoRI sites. Antibodies against 6His (Novagen), GST (Santa Cruz Biotechnology), the HA epitope (Biolegend), and α-tubulin (Sigma-Aldrich), were obtained from the indicated sources. Antibodies against RPN1 and PBA1 were used as previously described (Yu and Hua, 2022). Upon incubation with primary antibodies, each blot was further incubated with either goat anti-rabbit or goat anti-mouse secondary antibodies conjugated with horseradish peroxidase (SeraCare), and then visualized with SuperSignal West Pico Chemiluminescent Substrate or SuperSignal West Femto Maximum Sensitivity Substrate (Thermo Fisher Scientific), as according to the manufacturer’s instructions. Chemiluminescent signals on the immunoblot were scanned using an Azure 600 western blot imaging system (Azure Biosystems).

### Measurement of H_2_O_2_ production in root tips

*KRAB* and *P1-12* seedlings were germinated and grown in liquid 1/2 MS media supplemented with 0, 0.025, and 0.25 μM Dex for 4 d with slow shaking (100 rpm) at 23°C under continuous white light (120 μmol m^-2^ s^-1^). Upon treatment, 10 seedlings of each sample were selected and transferred separately into an Eppendorf tube containing 1 mL liquid 1/2 MS medium freshly mixed with 2 μM CM-H2DCFDA (Molecular Probes) for 10 min with gentle agitation. Fluorescence signals were observed under a Nikon Eclipse E600 epifluorescence microscope and recorded with Spot 5.2 software using the same exposure settings. Average fluorescence intensity in a fixed area (0.1 mm x 0.25 mm) that includes ~ 0.2 mm of a root tip was measured using ImageQuant5.2 (GE Healthcare).

## Results

### Reduction of *MORF2* transcripts by iCRISPRi inhibits seedling growth

CRISPRi applies a deactivated Cas9 (dCas9) fused with a transcriptional repressor to inhibit transcription of a target locus that is recognized by co-expressed sgRNAs (**Figure 1A**; Gilbert et al., 2013). Its effectiveness in plants has not been well documented (Karlson et al., 2021). To explore this technology, we adapted the *dCas9-KRAB* repressor from Gilbert et al (2014) and fused it with a Dex-inducible promoter (*Dex_p_*) in pTA7002 (Aoyama and Chua, 1997). Given the strong CRISPRi activity obtained within -50 to +300 nucleotide (nt) of the transcription start site (TSS) of a gene in human cells (Gilbert et al., 2014), we designed three *sgRNA* genes, *P1*, *P2*, and *M1*, containing a protospacer targeted to a similar region of the *MORF2* locus (**Figure 1B**; **Table S2**; Xie et al., 2014). We raised 4, 9, and 4 independent T4 homozygous transgenic lines that were transformed with a dual expression construct carrying *Dex_p_*:*dCas9-KRAB*::*U6-26_p_:P1*, *P2*, and *M1*, respectively. As a negative control, we obtained 6 independent lines stably transformed with *Dex_p_*:*dCas9-KRAB* alone. Hereafter, we referred them as *P1*, *P2*, *M1*, and *KRAB* transformants. After screening seedlings grown on 1/2 MS medium containing 5 μM Dex under LD, we found severe growth inhibition in three *P1* independent lines but only mild or nonsignificant phenotypes in *P2* and *M1* transformants, respectively. As a negative control, *KRAB* grew as normally as WT (**Figures 1C**, **S1A**). Therefore, a protospacer close to the TSS region is likely required for suppressing the transcription of *MORF2*.

To demonstrate iCRISPRi-mediated *MORF2* expression suppression, we first found by both sqRT-PCR and immunoblot analyses the expression induction of *dCas9-KRAB* in three *P1* transformants and *KRAB* upon Dex treatment. Neither protein bands nor RT-PCR products of *dCas9-KRAB* transgene were identified in WT or when transgenic seedlings were treated with DMSO only (**Figures 1D, E**). Consistent with the expression induction of *dCas9-KRAB*, we identified using anti-MORF2 polyclonal antibodies that the abundance of two protein species corresponding to the sizes of full-length (fl-) and truncated (t*-*) versions of MORF2 was apparently reduced in three *P1* seedlings treated with 5 μM Dex but not in other samples (**Figure 1F**). Additional qPCR confirmed significant reduction of *MORF2* transcripts in these samples as well (**Figure 1G**). While we detected a clear band of dCas9-KRAB by anti-HA antibodies in *P2* and *M1* seedlings treated with 5 μM Dex, the reduction of MORF2 was minor, suggesting that their weak phenotypes are likely due to the low efficiency of sgRNAs (**Figure S1B**).

### *P1* sgRNA is specific to *MORF2*

To rule out the possibility of off-target suppression by *P1* sgRNA, we analyzed the expression of six top off-target candidates (**Figure S2A**). Applying a transcriptome-wide analysis (see below), we only found *MORF2* with a greater than 2-fold expression reduction in *P1-12* treated with 0.25 μM Dex. The expression of off-targets maintained either no changes, undetectable, or upregulated in *P1-12* (+0.25 μM Dex) (**Figures S2B, C**). Therefore, the *P1* sgRNA does not likely bind to its putative off-target loci in *P1-12*.

To further test the specific expression inhibition of *MORF2*, we crossed *P1-12* with a *35S:MORF2-YFP* overexpression transgenic line to examine whether the reduced growth of *P1-12* upon 0.25 μM Dex treatment could be rescued. We also crossed *P1-12* with a *35S:HA-YFP* transgenic plant as a control. Because homozygous *MORF2* overexpression plants often induce co-suppression of *MORF2* and lead to variegation phenotypes not only in early seedlings but also to all aerial tissues in adult plants (**Figure S2D**), only F1 plants were analyzed. We detected no growth differences among the three genotypes when seedlings were treated with DMSO only. However, upon 0.25 μM Dex treatment, both primary root growth and lateral root formation were suppressed in *P1-12*. Interestingly, this suppression was completely restored in the F1 plants of *P1-12 x 35S:MORF2-YFP* (**Figures S2E, F**), suggesting that *MORF2-YFP* complements the expression suppression of the endogenous *MORF2*. We also observed a partial growth recovery in the F1 plants of *P1-12 x 35S:HA-YFP* likely due to a 50% reduction of *dCas9-KRAB* and *P1 sgRNA* alleles. Hence, *P1* sgRNA is specific to *MORF2*.

### MORF2 reduction suppresses plant growth in addition to affecting RNA editing

The development of iCRISPRi*-*mediated transcription suppression of *MORF2* allowed us to modulate its endogenous products in a controlled manner. Consequently, we can quantify the influence of MORF2 on RNA editing and plant growth. As a control, we also characterized a new *morf2-2* T-DNA insertion mutant allele to study the severest growth and developmental impact by loss-of-function of *MORF2* (**Figures 1B, F**). In addition to previously discovered albino and RNA-editing deficiency phenotypes in *morf2-1* (Takenaka et al., 2012), *morf2-2* showed retarded embryo development and disrupted embryo structures that led to varied seedling morphology upon germination (**Figure S3**).

Because *ndhB* mRNA has the severest editing errors in *morf2-1* and carries the highest number of RNA-editing sites among chloroplast mRNAs (Takenaka et al., 2012), we first compared RNA-editing efficiencies in its five codons that encode the 50th, 156th, 196th, 277th and 419th amino acids in 7-d-old LD-grown *P1-12* seedlings treated with different concentrations of Dex using a Sanger sequencing approach. While we detected approximately 25, 20, 10, 75, and 50% C-to-U editing efficiencies for the five sites in 7-d-old LD-grown *P1-12* seedlings treated with 5 μM Dex (**Figure 1H**), the editing efficiencies were either as normal as WT in *P1-12* seedlings treated with 0.25 μM Dex or fully abolished to 0% in *morf2-1* (**Figures 1H**, **S3E**, respectively), indicating a MORF2 dosage-dependent RNA-editing phenotype. Consequently, fewer RNA-editing errors were observed in *P1-10* and *P1-54* upon 5 μM Dex treatment due to a weaker suppression of *MORF2* (**Figures 1F–H**, **S1C**). No RNA editing errors were identified in WT and *KRAB* when treated with 5 μM Dex (**Figures 1H**, **S4**), further confirming the specificity of *P1* sgRNA to the *MORF2* locus.

We then examined the growth of 8-d-old *P1-12* seedlings grown vertically under LD on 1/2 MS media containing different concentrations of Dex. Surprisingly, we observed significant reductions of primary root length and density of lateral roots of its seedlings treated with Dex at a concentration as low as 0.025 μM, 200-fold below the concentration that caused detectable RNA-editing errors by the Sanger sequencing approach (**Figures 2A–C**). Interestingly, the expression induction of *dCas9-KARB* and suppression of *MORF2* were detected in accordance with seedling growth inhibition (**Figures 2D, E**). This result was inconsistent with the well-known RNA-editing function of MORF2 (Yuan et al., 2022). To carefully examine this discrepancy, we developed a transcriptome-wide analysis of chloroplast RNA-editing efficiency. Based on the counts of thymine and cytosine nucleotides present in reads aligned with a specific editing site, we were able to precisely decipher the editing efficiencies of all 33-known chloroplast RNA-editing sites (Takenaka et al., 2012; **Table S3**). Interestingly, three and eight sites had significant changes of C-to-U editing efficiency in *P1-12* (+0.025 μM Dex) and *P1-12* (+0.25 μM Dex), respectively, compared to *P1-12* (+DMSO). However, as a control, only one site showed a significant but small change of the editing efficiency in *KRAB* (+0.25 μM Dex) compared to *KRAB* (+DMSO) (*P* < 0.05, Fisher’s exact test with Bonferroni adjustment). Therefore, this digital data analysis better reflects the response of chloroplast RNA-editing machinery to the expression reduction of *MORF2*.

### MORF2 negatively regulates stress response

**Figure 3:**
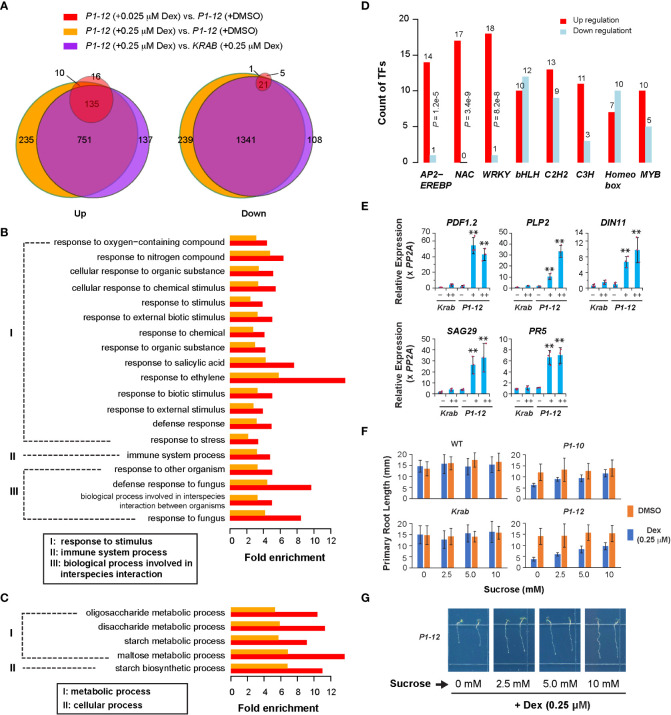
Transcriptome analysis revealed a role of *MORF2* in regulating plant stress defense. **(A)** Overlap of SDEGs in three indicated pairs of transcriptomes suggests a little impact of *dCas9-KRAB* overexpression but a large impact of *MORF2* reduction on transcriptome changes in *P1-12*. A gene was retained for comparison if its Counts Per Million (CPM) was above 2 in at least nine samples from two replicates each of *P1-12* (+DMSO), *P1-12* (+0.025 μM), *P1-12* (+0.25 μM), *KRAB* (+DMSO) and *KRAB* (+0.25 μM). **(B, C)** Enrichment of biological processes in up **(B)** and downregulated **(C)** SDEGs of *P1-12* seedlings treated with 0.025 μM (red bars) and 0.25 μM (orange bars) Dex demonstrated upregulation of stress response. **(D)** Overrepresentation of three major stress responsive transcription factor families, *APETALA2/ETHYLENE-RESPONSIVE ELEMENT BINDING PROTEIN* (*AP2-EREBP*), *NO APICAL MERISTEM/ARABIDOPSIS TRANSCRIPTION ACTIVATION FACTOR/CUPSHAPED COTYLEDON* (*NAC*), and *WRKY*, in upregulated SDEGs of *P1-12* seedlings treated with 0.25 μM Dex. *P* values indicated were calculated using Fisher’s exact test. **(E)** qPCR verification of five stress defensive genes. Relative expression of a target gene is normalized to one of the three *KRAB* biological replicates treated with DMSO. Bars and the maroon dots are described as in **Figure 1G**. Double asterisks represent a statistically significant upregulation of an indicated gene in *P1-12* seedlings treated with Dex compared to those treated with DMSO only (Student’s *t*-test, *P* < 0.01). –: DMSO; +: 0.025 μM Dex; ++: 0.25 μM Dex. **(F)** Sucrose promotes root growth of light-grown *P1-10* and *P1-12* seedlings only when treated with 0.25 μM Dex. Each bar represents mean ( ± SD) of 20 to 24 seedlings analyzed. WT and *KRAB* seedlings were included as negative controls. **(G)** Representative images of *P1-12* seedlings grown on 0.25 μM Dex-containing 1/2 MS media supplemented with different concentrations of sucrose.

To tackle early gene expression regulatory pathways involving MORF2, we analyzed the transcriptome changes of *P1-12* in response to the gradient expression reduction of *MORF2*. Through RNA-Seq analysis, we identified 161 and 1,131 upregulated SDEGs (≥ 2-fold changes, FDR (false discovery rate) < 0.01%), and 27 and 1,602 downregulated SDEGs, in 8-d-old LD-grown *P1-12* seedlings upon 0.025 and 0.25 μM Dex treatments, respectively, in comparison to the control treated with DMSO (**Figures 2A**, **3A**). Among them, 145 and 22 up and downregulated SDEGs (90.1 and 81.5%, respectively) from *P1-12* (+0.025 μM Dex) are included in *P1-12* (+0.25 μM Dex), suggesting that they are the early responsive genes to *MORF2* reduction (**Figure 3A**).

Similarly, 1,023 and 1,470 SDEGs were detected to be up and downregulated, respectively, in *P1-12* (+0.25 μM Dex) when compared with *KRAB* (+0.25 μM Dex) (**Figure 3A**). Among these, 886 and 1,362 (86.6 and 92.7%, respectively) were overlapped with the corresponding up and downregulated SDEG groups that were identified above (**Figure 3A**). Importantly, 98.2% overrepresented biological process (BP) GOs (all 79 upregulated and 198 out of 203 downregulated) in *P1-12* (+0.25 μM Dex) compared to *KRAB* (+0.25 μM Dex) were commonly found in *P1-12* (+0.25 μM Dex) compared to *P1-12* (+DMSO) (**Tables S4**, **5**). Hence, the influence of free dCas9-KRAB on the transcriptome changes in *P1-12* (+0.25 μM Dex) is negligible. Hereafter, we referred to the SDEGs of *P1-12* (+0.25 μM Dex) as compared with *P1-12* (+DMSO).

Given the growth inhibition phenotype (**Figures 2A–C**), the small group of SDEGs in *P1-12* (+0.025 μM Dex) likely reflects the early pathways in response to MORF2 reduction. Interestingly, all its 19 overrepresented upregulated BP GO pathways (Fisher’s exact test, *P* < 1e-10) were also significantly upregulated in *P1-12* (+0.25 μM Dex). Given their ancestor pathways in response to stimulus, immune system process and interspecies interaction, we hypothesized that reducing MORF2 in *P1-12* upregulated stress defense processes (**Figure 3B**). Five GO pathways involved in carbohydrate metabolism were commonly identified in the downregulated SDEGs of *P1-12* (+0.025 μM Dex) and *P1-12* (+0.25 μM Dex) despite a less statistically significant level (Fisher’s exact test, *P* < 0.01) applied in the former group due to its small size (**Figures 3A, C**). To rule out the possibility of a background change of GO pathways in *P1-12* (+0.025 μM Dex) due to a general effect of Dex treatment, we also identified 80 and 5 SDEGs that are up and down regulated in *KRAB* (+0.25 μM Dex) compared with *KRAB* (+DMSO). However, all 19 overrepresented upregulated BP GO pathways commonly found in *P1-12* (+0.025 μM Dex) and *P1-12* (+0.25 μM Dex) were not discovered at the 1e-10 *P-value* cutoff (Fisher’s exact test) in the 80 upregulated SDEGs of *KRAB* (+0.25 μM Dex) (**Table S6**). Similarly, the only 5 down-regulated SDEGs do not show any enrichment of GO pathways involved in carbohydrate metabolism either (**Table S7**).

The identification of significantly upregulated stress defensive pathways in *P1-12* (+0.025 μM Dex) and *P1-12* (+0.25 μM Dex) explains the growth suppression according to the growth-defense tradeoff model (Huot et al., 2014). To further demonstrate the upregulation of plant defense responses in *P1-12* upon Dex treatment, we 1) investigated the differential enrichment of all known transcription factor families, 2) analyzed the activity of senescence-associated genes (*SAGs*) as described in Garapati et al. (2015), 3) verified the expression of five selected stress responsive genes by qPCR, and 4) examined growth response to sucrose.

From a list of 52 transcription factor families including in total 1,851 annotated loci (Davuluri et al., 2003), we discovered that 8 families were overrepresented in the SDEGs of *P1-12* (+0.25 μM Dex). Among these, three well-known stress responsive families, *AP2-EREBP*, *NAC*, and *WRKY*, were discovered to be overrepresented in the upregulated SDEGs of *P1-12* (+0.25 μM Dex) (**Figure 3D**). Consistently, five selected genes involved in plant defense and senescence were detected by qPCR to be significantly upregulated in *P1-12* upon Dex treatment as low as 0.025 μM (**Figure 3E**). To support a notion of stress induced senescence and starvation (Mahmood et al., 2016; Sade et al., 2018), we first identified a significant enrichment of *SAGs* in the SDEGs of *P1-12* (+0.25 μM Dex) (**Table S8**). Unsurprisingly, 43 out of 46 enriched *SAGs* are upregulated (**Table S9**). Through germinating and growing seedlings on 1/2 MS media supplemented with different concentrations of sucrose, we found that addition of sucrose up to 10 mM alleviated the primary root growth suppression of *P1-10* and *P1-12* by 0.25 μM Dex treatment. This effect was not significant in WT and *KRAB* seedlings under both Dex and DMSO treatments (**Figures 3F, G**). Collectively, this transcriptome analysis suggests that MORF2 negatively modulates stress response beyond its role in RNA editing.

### MORF2 regulates different levels of chloroplast RS

**Figure 4:**
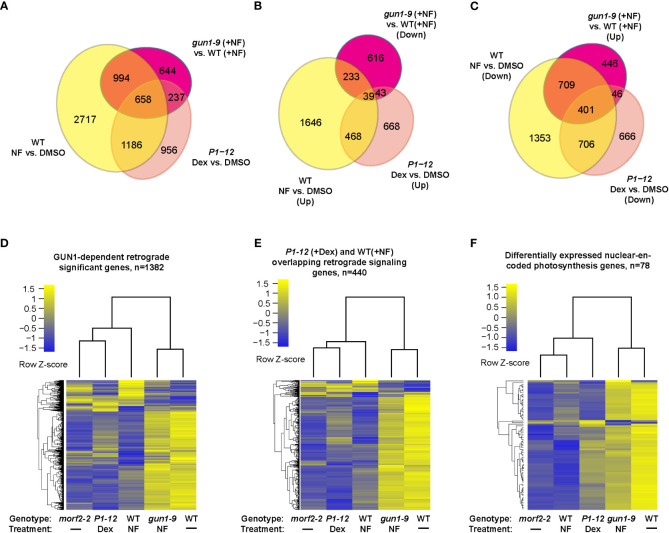
Cross experimental comparison demonstrates MORF2 as an effector of RS in chloroplasts. **(A)** Overlap of SDEGs resulting from MORF2 reduction in *P1-12* (+0.25 μM Dex) with known SDEGs involved in RS either in WT (Col6-3; +NF) or in *gun1-9* (+NF). The Venn diagram shows the overlap of SDEGs identified in *P1-12* (+0.25 μM Dex), WT (+NF), and *gun1-9* (+NF) compared to the corresponding controls indicated. A gene was retained for comparison if its CPM was above 0.5 in at least 11 samples from two replicates each of *P1-12* (+DMSO), *P1-12* (+0.25 μM), WT (+DMSO), WT (+NF), *gun1-9* (+NF), and *morf2-2*. **(B, C)** Identifying the group of known GUN1-dependent RS genes and its overlap with up and downregulated SDEGs in *P1-12* (+0.25 μM Dex). Shown are two Venn diagrams indicating the overlap of up or downregulated SDEGs from three different transcriptome pairs compared. **(D–F)** *P1-12* (+0.25 μM Dex) show similar and opposite expression patterns to WT (+NF) and *gun1-9* (+NF), respectively, for GUN1-dependent **(D)**, *P1-12* (+0.25 μM Dex)/WT (+NF) overlapping **(E)** retrograde signaling genes, and differentially expressed nuclear-encoded photosynthesis genes **(F)**. The two-dimensional clustering diagrams were created based on row Z-scores of normalized expression data (CPM) of each expressed gene (>0.5 CPM) from indicated samples.

Further GO analysis on the downregulated SDEGs of *P1-12* (+0.25 μM Dex) identified a significant enrichment of BP pathways related to photosynthesis, tetrapyrrole metabolism and pigment biosynthesis, all of which are characteristics related to RS (**Table S5**). Through comparison, we identified that 1,844 (60.7%) and 895 (29.5%) SDEGs of *P1-12* (+0.25 μM Dex) were also significantly differentially expressed in NF-treated WT (Col6-3) and *gun1-9* seedlings, respectively (**Figure 4A**; Zhao et al., 2019). We sorted out a group of 1,382 GUN1-dependent RS genes that are regulated in opposite in WT (+NF) and *gun1-9* (+NF) (**Figures 4B, C**). Interestingly, their expression patterns in *P1-12* (+0.25 μM Dex), particularly those overlapped with WT (+NF), had similar and opposite directions as they were in WT (+NF) and *gun1-9* (+NF), respectively (**Figures 4D, E**). Consistent with reduction of *MORF2* in *P1-12* (+0.25 μM Dex), those genes showed a similar expression pattern as did they in *morf2-2*. Further comparing *P1-12* (+0.25 μM Dex), *morf2-2*, and WT (+NF), we identified gradient expression changes, in which the GUN1-dependent RS genes showed higher fold-changes in *morf2-2* and WT (+NF) than did they in *P1-12* (+0.25 μM Dex) (**Figure 4D**). This feature is even more clear for the nuclear-encoded photosynthesis genes (**Figure 4F**; **Table S10**).

### Early RS pathways mediated by MORF2 suppress skotomorphogenesis

**Figure 5:**
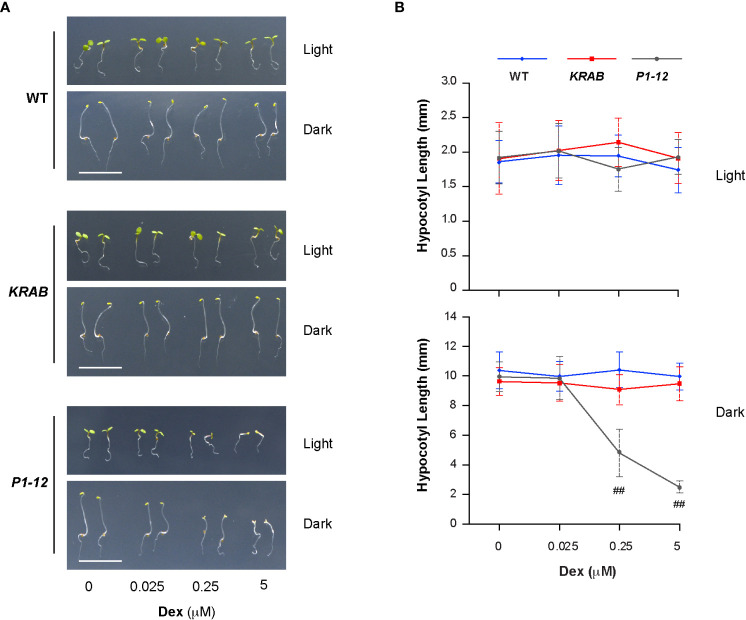
Reduction of MORF2 in *P1-12* upon Dex treatment inhibits skotomorphogenesis. **(A)** Representative seedlings grown for 3 d under LD or in dark on media containing different concentrations of Dex. Bar = 1 cm. **(B)** Effect of different concentrations of Dex on hypocotyl elongation. Seedlings were grown for 3 d as in **(A)** before measurement. Double hash tags identify points with significant reductions of hypocotyl length in *P1-12* than those in WT and *KRAB* (Student’s t test; *P* < 0.01). Each data point represents mean (± SD) of 20 replicates.

Lincomycin-induced RS suppresses photomorphogenesis (Martin et al., 2016; Gommers et al., 2021). Given the activation of RS in *P1-12* (+0.25 μM Dex), we questioned whether *P1-12* (+0.25 μM Dex) had altered morphogenesis. Since exogenous sucrose weakened the influence of lincomycin-induced RS (Martin et al., 2016), it was also eliminated hereafter. Upon 3 d after germination (DAG), we saw that WT, *KRAB*, and *P1-12* grew similarly under LD except for reduced cotyledon areas of *P1-12* when treated with ≥0.25 μM Dex (**Figure 5A**). However, we unexpectedly found opened cotyledons and shortened hypocotyls in dark-grown *P1-12* seedlings upon ≥0.25 μM Dex treatments (**Figures 5A, B**), indicating suppression of skotomorphogenesis by MORF2-mediated RS.

### Reducing MORF2 compromises ethylene signaling

**Figure 6:**
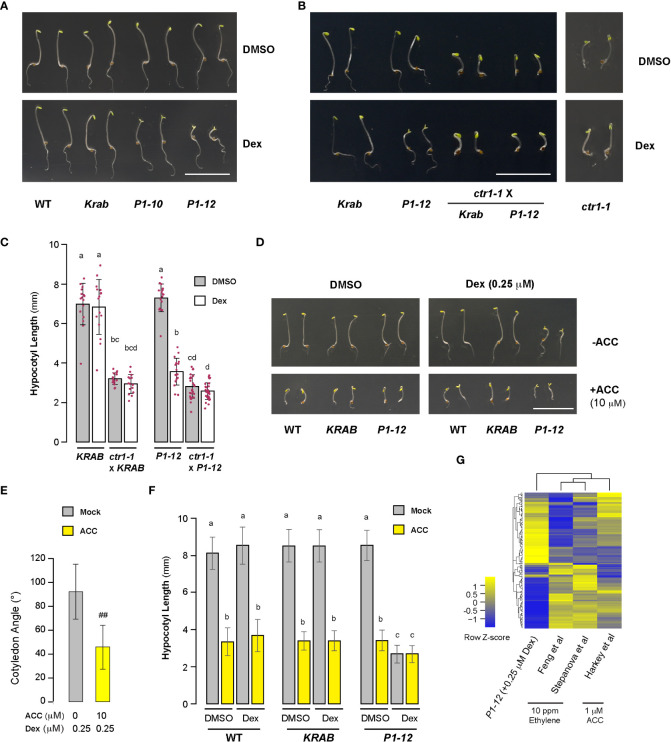
Skotomorphogenesis is partially suppressed by compromised ethylene signaling in *P1* seedlings upon Dex treatment. **(A, B)** Representative images of seedlings from indicated genotypes grown for 3 d in dark on media containing DMSO or 0.25 μM Dex. Bar = 1 cm. Growth images of *ctr1-1* were included to show its insensitivity to Dex treatment. **(C)** Quantitative analysis of hypocotyl length of seedlings as shown in **(B)** suggests that *MORF2* impacts ethylene signaling upstream of CTR1. Each bar herein as well as in **(E)** and **(F)** represents mean ± SD. The data points of replicates are indicated with maroon dots (n=16 except for *ctr1-1* x *P1-12*, which has 28 replicates). Different letters on the top of each bar indicate significant differences (Kruskal-Wallis test followed by Dunn’s test with Benjamini-Hochberg multiple testing correction, *P* < 0.05). **(D)** Images of representative seedlings grown for 3 d in dark on media supplemented with indicated combinations of DMSO, 0.25 μM Dex, and 10 μM ACC. **(E)** ACC treatment partially suppressed cotyledon opening of *P1-12* (+0.25 μM Dex) (##: Student’s *t*-test, *P* < 0.01; n=71 and 64 for mock and ACC treatments, respectively). **(F)** The hypocotyl length of *P1-12* (+0.25 μM Dex) is insensitive to ACC treatment. Different letters indicate significant differences as in **(C)** (n=72). **(G)** Comparison of expression changes of 128 “gold standard” genes involved in ethylene signaling among the indicated studies. *P1-12* (+0.25 μM Dex) is described as in **Figure 3A**. Studies from Feng et al. and Stepanova et al. were 6 and 3-d-old light-grown seedlings treated with 10 ppm ethylene, respectively, and that from Harkey et al. was 5-d-old light-grown seedlings treated with 1 μM ACC. The relative expression changes (log2(FC)) of “gold standard” genes for the last three studies were retrieved from Harkey et al. (2019) and used to compare with their changes in *P1-12* (+0.25 μM Dex). The two-dimensional clustering diagrams were created according to row Z-scores.

The unexpected discovery of open cotyledons in dark-grown *P1-12* (+0.25 and 5 μM Dex) seedlings led us to hypothesize that MORF2-mediated RS suppresses ethylene signaling given its role in plant skotomorphogenesis (Leivar and Quail, 2011; Zdarska et al., 2015). To test this, we found a similar cotyledon opening phenotype in dark-grown *P1-10* (+0.25 μM Dex) seedlings. In addition, the cotyledon opening of *P1-12* (+0.25 μM Dex) was suppressed by *constitutive triple response 1* (*ctr1-1*) (**Figures 6A, B**). Hence, suppression of skotomorphogenesis is neither due to transgenic position effect nor blocking ethylene signaling downstream of CTR1. Interestingly, addition of 10 μM ACC, the precursor of ethylene, significantly reduced but did not completely close cotyledon angles (**Figures 6D, E**), suggesting that MORF2-mediated RS acts on ethylene biosynthesis. Both elimination of *CTR1* function and ACC treatment did not change the hypocotyl length of *P1-12* (+0.25 μM Dex) (**Figures 6C, F**), implying that non-ethylene pathways also impact its morphology changes in dark.

Compromised ethylene signaling seemed to be contradictory to the upregulation of “response to ethylene” (GO:0009723) pathway in 8-d-old LD-grown *P1-12* (+0.25 μM Dex) seedlings (**Figure 3B**). To address this discrepancy, we investigated the expression changes of a “gold standard” group of 139 ethylene-regulated genes (Harkey et al., 2019). In total, 128 were found to be differentially expressed in *P1-12* (+0.25 μM Dex). Through comparing their expression responses to either ethylene or ACC treatments (Harkey et al., 2019), we identified a strikingly opposite regulation in *P1-12* (+0.25 μM Dex) (**Figure 6G**; **Table S11**). After extracting 25 SDEGs involved in GO:0009723 from *P1-12* (+0.025 μM Dex) (**Table S12**), we found none from the “gold standard” group but three *ETHYLENE RESPONSIVE FACTORs* (*ERF8*, *ERF15*, and *ERF59*), which could be regulated in an ethylene independent manner, such as ROS-activated MITOGEN-ACTIVATED PROTEIN KINASE (MPK) pathways (Meng et al., 2013). Hence, the upregulation of GO:0009723 in *P1-12* (+0.25 μM Dex) is not likely through ethylene signaling.

### MORF2-mediated RS inhibits *PIF* expression

**Figure 7:**
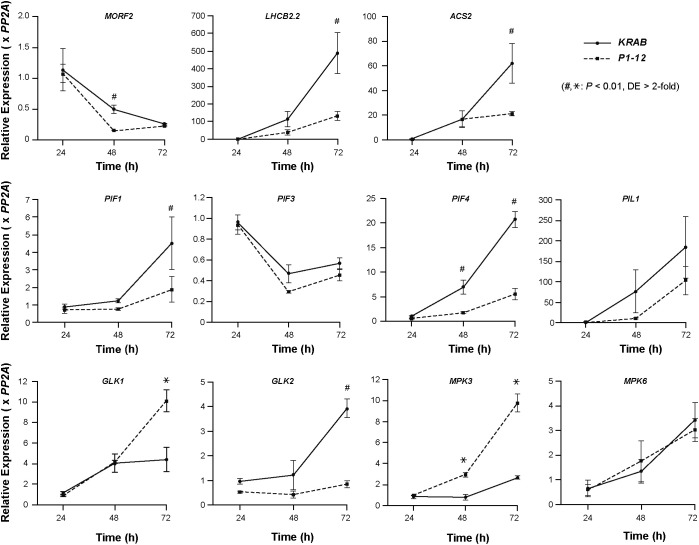
Early RS induced by *MORF2* reduction suppresses *PIFs* but activates *GLK1* and *MPK3* expression. Upon stratification on 1/2 MS medium supplemented with 0.25 μM Dex in dark at 4°C for three days, *KRAB* and *P1-12* seeds were light-treated for 6 hours followed by germination in dark for 24, 48, and 72 h. Either ruptured seeds (24 h) or etiolated seedlings with seed coat (48 h and 72 h) were harvested for qPCR analysis. Relative expression of each indicated gene was normalized to one of the three *KRAB* biological replicates at 24 h post germination. *PP2A* was used as reference standards. Each data point represents mean ( ± SD) from three biological replicates, each with three technical replicates. # and * represent a statistically significant reduction and upregulation, respectively, of indicated gene in *P1-12* compared to *KRAB* (Student’s *t*-test, *P* < 0.01 and differential expression (DE) changes > 2-fold).

PHYTOCHROME INTERACTING FACTOR (PIF)s play a pivotal role in positively regulating skotomorphogenesis and interacting with ethylene signaling (Jeong et al., 2016; Paik et al., 2017). To search for additional factors contributing to the skotomorphogenic phenotypes of *P1-12* (+0.25 μM Dex), we compared by qPCR the expression of three *PIFs* (*PIF1*, *PIF3*, and *PIF4*) and their direct targets, including *PIF3-LIKE* (*PIL*)*1* and *GOLDEN2-LIKE* (*GLK*)*1* (Martin et al., 2016; Ueda et al., 2020), between 3-d-old dark-grown *P1-12* (+0.25 μM Dex) and *KRAB* (+0.25 μM Dex) (**Figure 7**). Consistent with suppressed skotomorphogenesis, we detected reduced expression of *PIFs*, including *PIF1* and *PIF4* whose expression was significantly lower in *P1-12* (+0.25 μM Dex) than in *KRAB* (+0.25 μM Dex). Consequently, *PIL1* and *GLK1*, which are positively and negatively regulated by PIFs in dark, were downregulated and upregulated, respectively, in *P1-12* (+0.25 μM Dex). *GLK2*, the homologue of *GLK1*, however, demonstrated a lower expression in *P1-12* (+0.25 μM Dex) than that in *KRAB* (+0.25 μM Dex). The opposite expression regulation of *GLK1* and *GLK2* further indicates the presence of yet-unidentified *GLK1*-specific pathways that positively contribute to the cotyledon opening both in dark-grown *P1-12* (+0.25 μM Dex) and lincomycin-treated light-grown seedlings (**Figure 5** and Martin et al., 2016, respectively).

To confirm the activation of RS and compromised ethylene signaling in dark-grown *P1-12* (+0.25 μM Dex) seedlings, we identified that one RS marker gene, *LHCB2.2*, and one *ACC SYNTHASE* (*ACS*) gene, *ACS2*, had a significantly lower expression in *P1-12* (+0.25 μM Dex) than in *KRAB* (+0.25 μM Dex) (**Figure 7**). Downregulation of *ACS2* could be also in part due to the reduction of *PIFs* since PIF4/5 have been discovered to positively regulate its expression (Song et al., 2018).

Unexpectedly, we found no significant difference of *MORF2* expression in 3-d-old dark grown seedlings (**Figure 7**). We therefore backtracked in 1- and 2-DAG seedlings. We identified a development-dependent upregulation of *PIF1*, *PIF4*, *PIL1*, *ACS2*, *LHCB2.2*, and *GLK2* in *KRAB* (+0.25 μM Dex) from 1-DAG to 3-DAG whereas this upregulation was significantly retarded in *P1-12* (+0.25 μM Dex). Contrastingly, the expression of *MORF2* declines more rapidly in *P1-12* (+0.25 μM Dex) than in *KRAB* (+0.25 μM Dex). In 2-DAG seedlings, *P1-12* (+0.25 μM Dex) had only 30.8% of the *MORF2* transcripts as detected in *KRAB* (+0.25 μM Dex). These findings suggested that a RS pathway was activated in early etiolated seedlings of *P1-12* (+0.25 μM Dex) due to rapid reduction of *MORF2*. Upon activation, it suppresses the expression of *PIFs*, at least *PIF1* and *PIF4* examined, which in turn upregulates GLK1 and downregulates ethylene signaling, thus promoting cotyledon opening. Of note, this activated RS also significantly upregulates the expression of *MPK3* but not *MPK6* in *P1-12* (+0.25 μM Dex) (**Figure 7**), which in part explained the activation of *ERFs* (**Table S12**).

### Antagonistic interaction between RS mediated by MORF2 and that induced by lincomycin

The opposite function in regulating cotyledon opening between RS mediated by MORF2 and that induced by lincomycin suggests their antagonistic interactions. To test this, we grew *P1-12* and *KRAB* seedlings on 1/2 MS medium supplemented with 0.25 μM Dex and lincomycin both in dark and in a constant dim light condition as previously described (Martin et al., 2016). As controls, we also treated the seedlings with NF and examined the skotomorphogenesis of *pifq* under the same treatments in dark.

**Figure 8:**
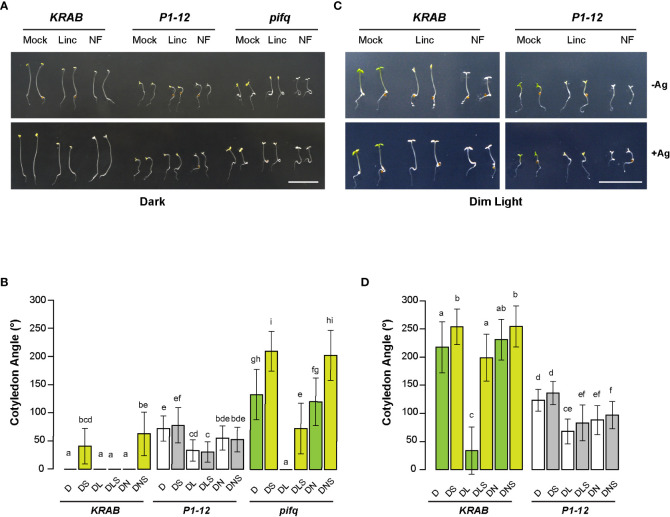
MORF2-dependent RS plays an antagonistic role to lincomycin-induced RS on plant photomorphogenesis. **(A)** Representative images of seedlings from indicated genotypes grown for 3 d in dark on media containing 0.25 μM Dex in the presence or absence of 5 μM AgNO3. Bar = 1 cm. **(B)** Quantitative analysis of cotyledon angle of seedlings shown in **(A)**. Different letters on the top of each bar indicate significant differences as analyzed in **Figure 6C** (n = 30-35). D: 0.25 μM Dex; L: 0.5 mM lincomycin; N: 5μM NF; S: 5 μM AgNO3. **(C)** Representative images of seedlings from indicated genotypes grown for 3 d under continuous dim light on media containing 0.25 μM Dex in the presence or absence of 5 μM AgNO3. Bar = 1 cm. **(D)** Quantitative analysis of cotyledon angle of seedlings shown in **(C)**. Different letters on the top of each bar and D, L, N, and S are indicated as in **(B)**.

Upon three days of germination in dark, we observed open cotyledons of *P1-12* (+0.25 μM Dex) and *pifq* (+0.25 μM Dex) seedlings grown under both mock (0.25 μM Dex only) and NF treatments (**Figure 8A**). However, their cotyledon opening responded differently to lincomycin treatment. While the cotyledon angles of *pifq* (+0.25 μM Dex) were closed by lincomycin treatment as previously reported (Martin et al., 2016), those of *P1-12* (+0.25 μM Dex) seedlings only reduced 54% (**Figures 8A, B**), suggesting that MORF2-mediated RS and lincomycin-induced RS antagonistically regulate cotyledon opening in dark.

Consistent with the previous discovery showing that lincomycin-induced RS enhances the ethylene pathway (Gommers et al., 2021), silver treatment opened the otherwise appressed cotyledons of lincomycin-treated *pifq* (+0.25 μM Dex) in dark. This enhancement along with the PIF pathways collaboratively retained the closure of cotyledons in dark-grown *KRAB* (+0.25 μM Dex) seedlings even after the silver treatment (**Figure 8B**). Contradictory, the impact of silver on the changes of cotyledon angles of dark-grown *P1-12* (+0.25 μM Dex) seedlings was negligible in all growth conditions (**Figure 8B**), further suggesting a compromised ethylene response in *P1-12* (+0.25 μM Dex).

The lack of silver sensitivity was also evident in light-grown *P1-12* (+0.25 μM Dex) seedlings (**Figures 8C, D**). While lincomycin-induced cotyledon closure was nearly inhibited by silver treatment in light-grown *KRAB* (+0.25 μM Dex) seedlings, only a non-significant increase was identified in sliver and lincomycin-treated *P1-12* (+0.25 μM Dex) seedlings. Consistent with the antagonistic interaction model, lincomycin treatment reduced cotyledon angles of *P1-12* (+0.25 μM Dex) seedlings significantly but not as severely as did it on *KRAB* (+0.25 μM Dex) seedlings (1.8-fold vs. 6.4-fold reductions, respectively). Of note, we observed no significant impact of NF on cotyledon angles of dark-grown *pifq* (+0.25 μM Dex) and light-grown *KRAB* (+0.25 μM Dex) seedlings. While the former was consistent with the previous study (Martin et al., 2016), the latter was not. We did not see any significant impact of NF on WT cotyledon angles either (**Figure S5**). This discrepancy could be caused by a mild impact of NF on photomorphogenesis and/or growth condition differences.

### High accumulation of ROS is concomitant with MORF2 reduction

**Figure 9:**
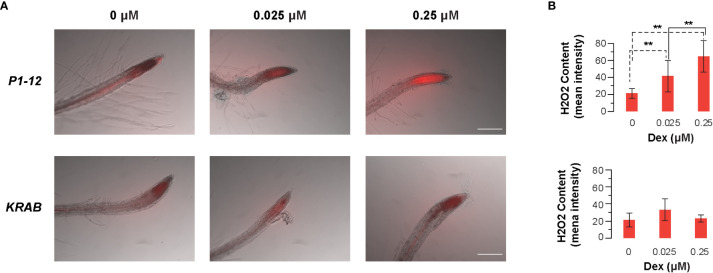
Dex-induced inhibition of *MORF2* through dCas9-KRAB-mediated transcriptional repression in *P1-12* increases accumulation of H_2_O_2_ in root tips. **(A)** Merged images of bright field (grayscale) and fluorescence (red pseudocolor) showing localization and accumulation of H_2_O_2_ at the root tip of 4-d-old *P1-12* and *KRAB* seedlings treated with different concentrations of Dex. H_2_O_2_ content was displayed by 2 μM CM-H2DCFDA for 10 min before microscopy examination. Fluorescence indicates the presence of H_2_O_2_. Scale bar = 100 μm. **(B)** Quantification of H_2_O_2_ content in root tips. To avoid the size verification of root tips, average fluorescence intensity of a fixed area (0.1 mm x 0.25 mm) that includes ~ 0.2 mm of root tip was measured using ImageQuant5.2 and used to reflect the relative H_2_O_2_ content. In total 10 seedlings were analyzed for each data point except for *KRAB* (+0.025 M Dex), in which 9 seedlings were analyzed. Fluorescence images of all the replicates are presented in **Figure S6**. **: P < 0.01 (Student's t-test, n=10).

To search for a potential downstream metabolic response to MORF2 reduction, we monitored the root tip hydrogen peroxide (H_2_O_2_) content in 4-d-old liquid-grown *P1-12* seedlings under constant light using CM-H2DCFDA (Wojtala et al., 2014). Upon staining, increasing concentrations of H_2_O_2_ became evidently detected in the root tip of *P1-12* seedlings upon 0.025 μM Dex treatment (**Figures 9A**, **S9A**). When Dex concentration increased to 0.25 μM, more than 3-fold higher of H_2_O_2_ content was detected in the root tip of *P1-12* seedlings than that without Dex treatment (**Figure 9B**). Consistent with the normal expression of *MORF2*, only a background level of H_2_O_2_ was detectable in the root tip of *KRAB* seedlings despite the Dex treatment (**Figures 9**, **S9B**). Hence, the increase of H_2_O_2_ in *P1-12* seedlings upon Dex treatment most likely resulted from the expression downregulation of *MORF2* but not from the ectopic expression of *dCas9-KRAB*. H_2_O_2_ has been known as one type of RS molecules (Exposito-Rodriguez et al., 2017). Its rapid upregulation upon *MORF2* transcriptional reduction in *P1-12* seedlings treated with Dex further supports a novel role of MORF2 in mediating RS.

## Discussion

In this study, we provided several lines of evidence demonstrating the complexity of RS pathways originated in chloroplasts and MORF2-mediated regulation of chloroplast development and functions. First, we developed a *dCas9-KRAB* inducible expression-based quantitative manipulation of endogenous genes in plants. To our best knowledge, this is the first report showing the application of CRISPRi in deciphering the function of a genetic hub gene. Second, our results uncovered a dosage-dependent (i.e., *MORF2* expression level) novel RS resource that plays an antagonistic role to lincomycin-induced RS in regulating plant morphogenesis. Third, we verified the roles of MORF2 in regulating both RS and RNA editing in chloroplasts.

### Application of iCRISPRi in quantitative reverse genetics studies

Because the *morf2*-1 and *morf2*-2 T-DNA null mutants showed multiple growth defects that are much severer than several other RNA editing mutants (**Figure S3**; Bisanz et al., 2003; Takenaka et al., 2012; Small et al., 2020), we thought that plants could be sensitive to the expression level of *MORF2*. To manipulate its expression, one could apply a post-transcriptional regulation such as RNA-interference or microRNA-mediated RNA degradation (Ostergaard and Yanofsky, 2004). Unfortunately, our overexpression analysis of *MORF2* discovered its strong co-suppression phenotype both in seedlings and adult plants (**Figure S2D**), which indicates that *MORF2* mRNA could be too susceptible to be quantitatively manipulated post-transcriptionally. Given the success of CRISPRi on gene expression regulation in human cells (Gilbert et al., 2014; Konermann et al., 2015), we explored this technology herein to avoid a potentially complex post-transcriptional regulation of *MORF2*. Through controlling the expression level of *dCas9-KRAB* repressor by a Dex-inducible promoter, we demonstrated our improved iCRISPRi method for quantitatively manipulating the transcription of *MORF2* (**Figures 1**, **2**). This new method provided a genetic tool for quantitative reverse genetics studies on hub genes in the future.

### Chloroplasts generate multiple RS pathways directing complex regulation of nuclear gene expression

Accumulating evidence indicates chloroplasts as a central organelle for stress sensing and response (Serrano et al., 2016; Crawford et al., 2018; Hu et al., 2020; Dopp et al., 2021; Li and Kim, 2022; Sierra et al., 2022). Given various types of stresses, chloroplasts most likely invoke different RS pathways to regulate the expression of nuclear genes in response to the stress signals. To date, multiple chloroplast metabolites, including intermediates from tetrapyrrole biosynthesis and MEP pathways, carotenoid derivatives, fatty acids, and ROS, have been proposed as chloroplast RS molecules (Dopp et al., 2021). While it is yet not clear about the signaling molecules involved in lincomycin and NF-induced RS pathways, several studies have demonstrated both overlapping and distinct responses triggered by these two chemicals. For example, the *gun* phenotype shown in NF-treated *MORF2* overexpression lines was not replicated by lincomycin treatment (Zhao et al., 2019). On the other hand, lincomycin-induced RS suppressed the constitutive photomorphogenic phenotype of *pifq* in dark whereas NF failed (Martin et al., 2016). Since lincomycin inhibits translation machinery in chloroplasts while NF impacts carotenoid biosynthesis (Ellis, 1970 and Frosch et al., 1979, respectively), it suggests that lesions of different chloroplast activities employ either distinct or different levels of RS pathways. Consistently, it was also proposed that acclimation response in chloroplasts involves time- and concentration-dependent composition of RS signal mix (Dietz, 2015; Chan et al., 2016). Therefore, it is not surprising to discover a MORF2 dosage-dependent RS pathway that plays an antagonistic role to the lincomycin-induced RS in plant morphogenesis regulation (**Figure 8**).

### MORF2 is a key RS effector with a large binding capacity

RS and RNA editing are two distinct gene expression regulatory pathways that influence the chloroplast proteome homeostasis. Their interactions had not been clear until the discovery of RNA-editing errors in NF-treated *gun1* mutant and *MORF2* overexpression plants (Zhao et al., 2019). It was proposed that RNA-editing errors might either perturb the expression of subunits for plastid-encoded RNA polymerase (PEP) or result in unfolded protein stress response in chloroplasts, hence, activating GUN1-dependent RS (Zhao et al., 2019). However, in our transcriptome-wide analysis, we did not find a significant defect of RNA-editing in all the known editing sites of the three PEP subunits in *P1-12* (+0.25 μM Dex) (**Table S3**). Instead, nine sites present in *ndhB*, *ndhD*, *ndhF*, *psbZ*, *rpl23*, and *rps14* showed perturbed RNA-editing efficiencies. Although we cannot rule out the possibility that the lethal phenotype of *morf2* is likely an accumulative effect of increasing RNA editing errors and RS response, we speculated that some defective growth phenotypes of *P1-12* (+0.25 μM Dex) might result from additional biochemical functions of MORF2. For example, the less than 15% reductions of RNA-editing efficiencies occurred in only five editing sites, including *ndhB* (two sites with 15.3 and 10.1% reductions), *psbZ* (one site with 9.8% reduction), and *rps14* (two sites with 5.1 and 4.0% reductions), might not have a profound growth effect. Consistent with our hypothesis, thirteen sites present in *accD*, *matK*, *ndhB*, *ndhD*, *ndhG*, *rpoB*, and *rps12* were found to have > 90% reductions of editing efficiencies in a T-DNA insertional mutant of *Organelle RRM protein 1*, *orrm1*. However, *orrm1* was shown to grow as normally as WT (Sun et al., 2013).

The essential role of MORF2 in RS could be attributed to its large array of binding partners in chloroplasts, such as MORF proteins (Zehrmann et al., 2015; Huang et al., 2019), PPR proteins involved in RNA-editing (Takenaka et al., 2012), GUN1 (Zhao et al., 2019), and multiple enzymes and regulators involved in tetrapyrrole biosynthesis (Zhang et al., 2014; Yuan et al., 2022). Its large binding capacity is consistent with its structural feature that can be divided into an N-terminal intrinsically disordered region and a peptidase inhibitor I9 domain-containing C-terminal region (Luo et al., 2017; Varadi et al., 2022). Both regions could be sticky to multiple interacting partners. Intrinsically disordered proteins have a one-to-many promiscuous binding capacity (Uversky, 2013), while peptidase inhibitor I9 members are known to function as molecular chaperones (Li et al., 1995). Interestingly, a recent work demonstrated that MORF2 possesses a holdase chaperon activity (Yuan et al., 2022). Therefore, we predicted that more MORF2-binding partners could be discovered in the future. Further supporting our discovery about the role of MORF2 in RS beyond RNA-editing, multiple MORF2-interacting enzymes and regulators involved in tetrapyrrole biosynthesis do not affect plastid RNA editing either (Yuan et al., 2022).

### A proposed model underlying the molecular function of MORF2

Given the holdase chaperon activity of MORF2 with a large binding capacity and the activation of RS pathways upon MORF2 reduction, we proposed that *MORF2* is a genetic hub gene for balancing plant growth and defense. Although the present study has not established a direct connection between ROS and RS in *P1-12* (+Dex), the rapid accumulation of ROS and upregulation of *MPK3* in *P1-12* (+Dex) indicate that ROS is one important type of metabolites downstream of MORF2 and that activation of MPK3 in cytoplasm relays the signal towards nucleus. The MORF2 dosage-dependent RS regulates not only nuclear genome-encoded chloroplast genes but also those associated with stress including three major groups of stress responsive transcription factors, *AP2-EREBPs*, *NACs*, and *WRKYs* (**Figures 3**, **4**). The finding of rapid reduction of *MORF2* transcripts upon development in normal dark-grown seedlings, such as *KRAB* (+0.25 μM Dex) (**Figure 7**), suggests that some MORF2-dosage dependent RS pathways are activated even in dark-grown WT seedlings. These RS pathways may help turn off skotomorphogenesis quickly upon the exposure of light. Its antagonistic role with lincomycin-induced RS pathways in plant morphogenesis further indicates the presence of a complex RS network. The former is activated in dark while the latter is involved in response to high light.

## Data availability statement

The datasets presented in this study can be found in online repositories. The names of the repository/repositories and accession number(s) can be found in the article/**Supplementary Material**.

## Author contributions

ZH designed and supervised the research. MY, PD, ZG, PY, and ZH performed experiments. ZH analyzed the data and wrote the article. All authors contributed to the article and approved the submitted version.
