# Supplementary material for: MORF2-mediated plastidial retrograde signaling is involved in stress response and skotomorphogenesis beyond RNA editing
Source: Front Plant Sci. 2023 Mar 28;14:1146922. doi: 10.3389/fpls.2023.1146922 (PMC10086144; doi:10.3389/fpls.2023.1146922)
Supplement: Supplementary file 1 [file DataSheet_1.pdf]

Supplementary Figure Legends

Figure S1 | Different effects of three *sgRNA* genes containing protospacers *P1*, *P2*, and *M1* in directing dCas9-KRAB-mediated transcriptional repression of *MORF2*. **(A)** Representative images showing seedlings of different genotypes (right panel) grown on 1/2 MS media supplemented with DMSO (left panel) or with 5 μM Dex (middle panel). *P1*, *P2*, and *M1* indicates plants transformed with *dCas9-KRAB* and a *sgRNA* gene containing *P1*, *P2,* or *M1* protospacer sequences, respectively. **(B)** Immunoblot analysis showing different reductions of both full-length (fl) and truncated (t) versions of MORF2 in *P1*, *P2*, and *M1* transgenic seedlings compared to WT and *KRAB*. All seedlings were grown on 1/2 MS medium supplemented with 5 μM Dex under LD for 7 d before analysis. Antibodies against HA were used to identify the protein abundance of dCas9-KRAB. Nearly equal protein loading was confirmed with anti-tubulin antibodies. The asterisk identifies an unknown cross-reacting species.

Figure S2 | *P1* protospacer is specific to *MORF2*. **(A)** List of six top off-target candidates of *P1* protospacer predicted by two programs as indicated. Red color highlights nucleotides in the sequence of an off-target that are different to the *P1* protospacer sequence. The "offTarget" and "Cas-OFFinder" programs were developed by Xie et al., 2017 and Bae et al., 2014, respectively. **(B)** Expression changes of five off-targets in *KRAB* and *P1-12* seedlings upon 0.25 μM Dex treatment. The expression was determined based RNA-Seq analysis as described in Fig. 3. **(C)** A metadata analysis indicates a specific expression of *AT2G33420* in flower, pollen, and silique tissues. **(D)** Representative images showing the variegation phenotypes of *35S:MORF2-YFP* at seedling (top panel) and bolting (bottom panel) stages. **(E, F)** The growth of *P1-12* is restored in F1 seedlings by crossing with *35S:MORF2-YFP* better than with *35S:HA-YFP*. (E) Representative images showing seedlings of indicated genotypes germinated and vertically grown under LD on 1/2 MS media supplemented with DMSO (top panel) or with 0.25 μM Dex (bottom panel) for 6 d. (F) Quantitative analysis of primary root length of seedlings shown in (E). The *P* values were calculated based on Student's *t*-test between indicated pairs of samples. The maroon dots indicate eight data points of replicates in each bar.

Figure S3 | T-DNA insertion mutation results in severe defects in embryo development of *morf2-2* in addition to RNA-editing errors. **(A)** Seed development is retarded in *morf2-2*. Pictured are siliques from self-fertilized wild-type and heterozygous *morf2-2* plants. White arrowheads indicate seeds with retarded development. **(B, C)** Images showing retarded embryo development of *morf2-2*. While the WT embryo has entered the late globular (B) or

torpedo (C) stages, the *morf2-2* embryo is still at the 16-cell dermatogen (B) or heart (C) stages, respectively. Abnormal shapes with enlarged cotyledon primordia of *morf2-2* embryo appear at the heart stage (C). **(D)** Abnormal development of cotyledon and true leaves of *morf2-2*. Images of cotyledons (1-3) were taken from ~4-d-old seedlings and that of true leaves were taken from 20-d-old seedlings grown on 1/2 MS medium under LD. The image of 4-d-old WT seedling was included as a control. **(E)** The *morf2-2* is deficient in C to U conversion for the four RNA-editing sites of *ndhB*. Blue arrow heads point the cytidine sites with abolished editing effects. Genetic codons containing an editing site are capitalized and numbered.

Figure S4 | Dex treatment does not cause RNA-editing errors in 7-d-old light-grown seedlings of both WT and *KRAB*. Five RNA-editing sites of *ndhB* were analyzed as in Fig. 1H. Genetic codons containing an editing site are capitalized and numbered.

Figure S5 | NF does not inhibit photomorphogenesis of WT seedlings grown under dim light. The seedlings were grown and treated as in Fig. 8C-D. The data points of replicates are indicated with maroon dots (n = 60-70). Different letters on the top of each bar indicate significant differences as analyzed in Fig. 6C. D: 0.25 μM Dex; M: 0.1% DMSO; L: 0.5 mM lincomycin; N: 5μM NF.

Figure S6 | Fluorescence images showing H_2O_2 accumulation in root tips of Dex-treated *P1-12* but not *KRAB* seedlings. Seedlings were grown under constant light in liquid 1/2 MS media supplemented with indicated concentrations of Dex for 4 d before examination under a Nikon E600 epifluorescence microscope. H_2O_2 content was detected by incubating the seedlings with 2 μM CM-H2DCFDA for 10 min. **(A)** Fluorescence images of root tips from *P1-12* seedlings treated with different concentrations of Dex. **(B)** Fluorescence images of root tips from *KRAB* seedlings treated as in (A).
