# Supplementary figures and images for: MORF2-mediated plastidial retrograde signaling is involved in stress response and skotomorphogenesis beyond RNA editing
Source: Front Plant Sci. 2023 Mar 28;14:1146922. doi: 10.3389/fpls.2023.1146922 (PMC10086144; doi:10.3389/fpls.2023.1146922)

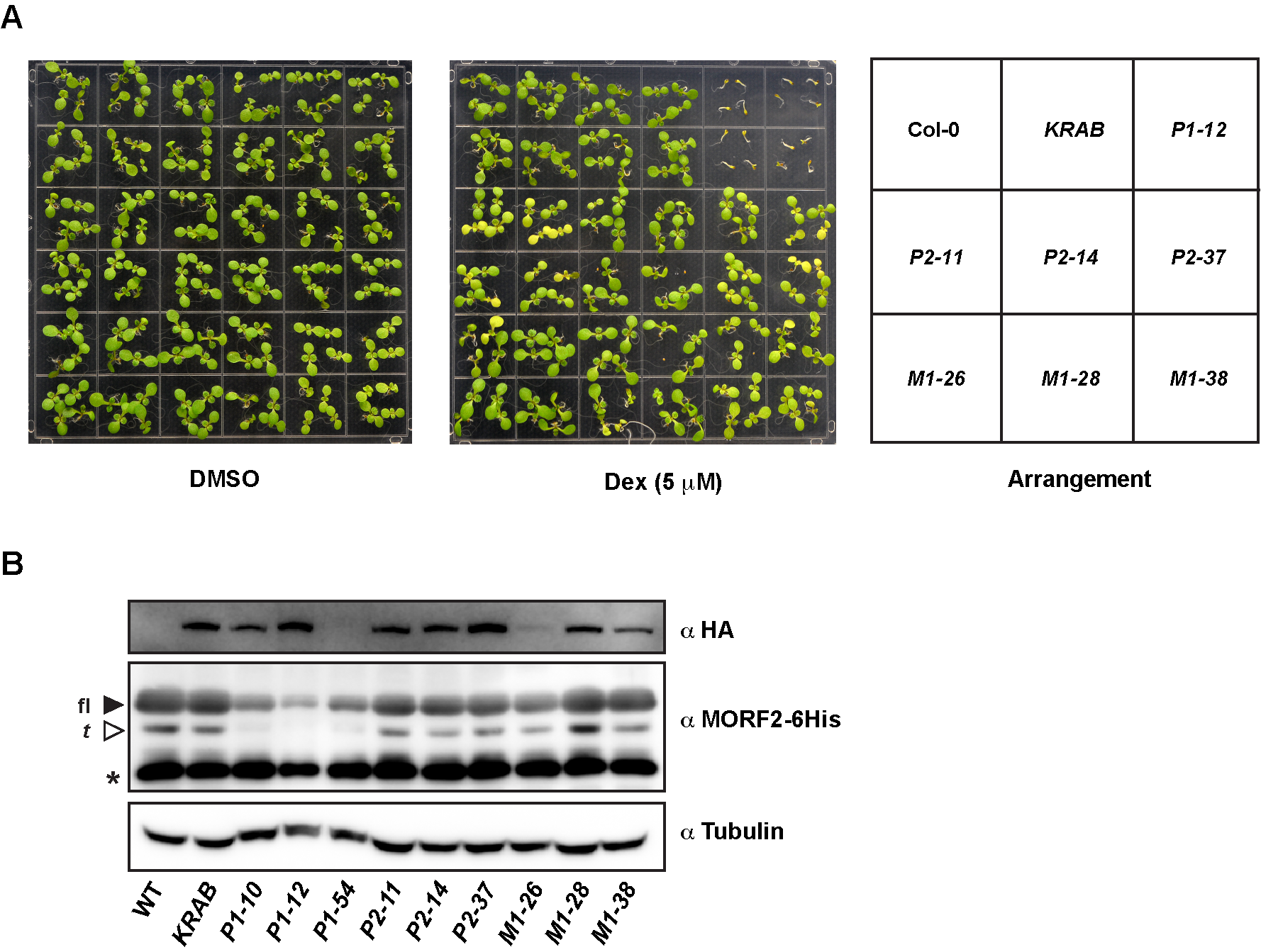

Supplement: Supplementary file 2 [file Image_1.tif]

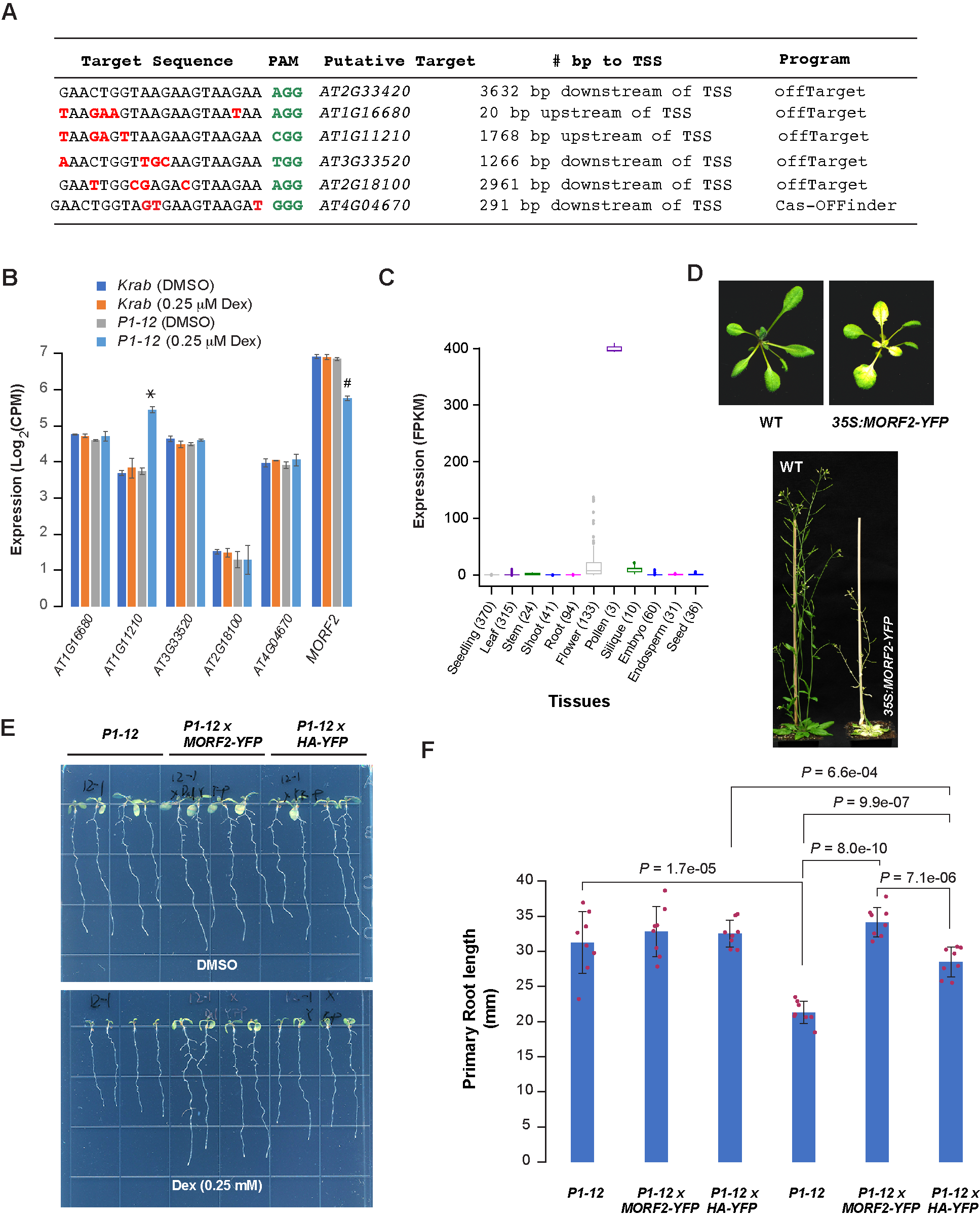

Supplement: Supplementary file 3 [file Image_2.tif]

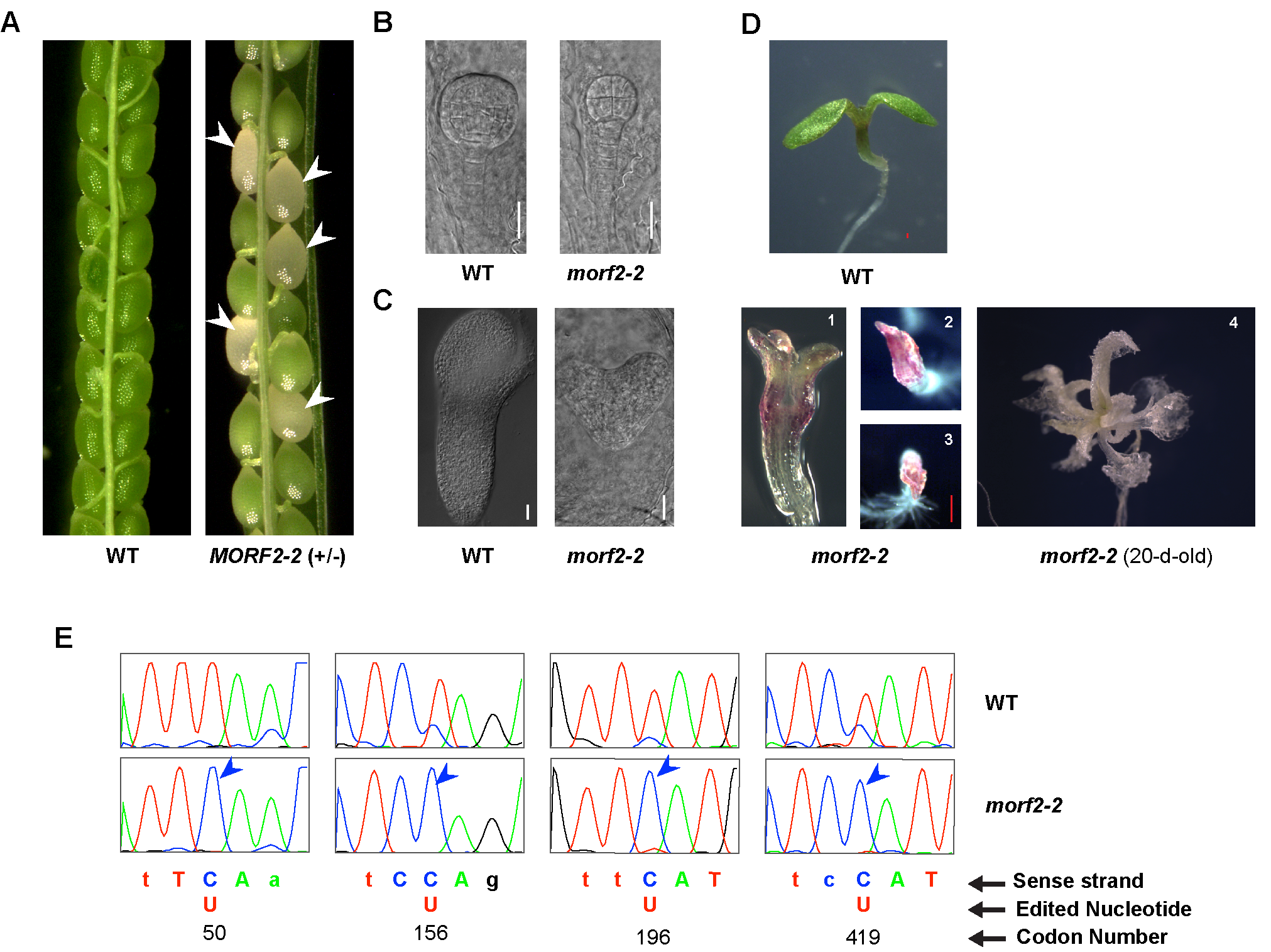

Supplement: Supplementary file 4 [file Image_3.tif]

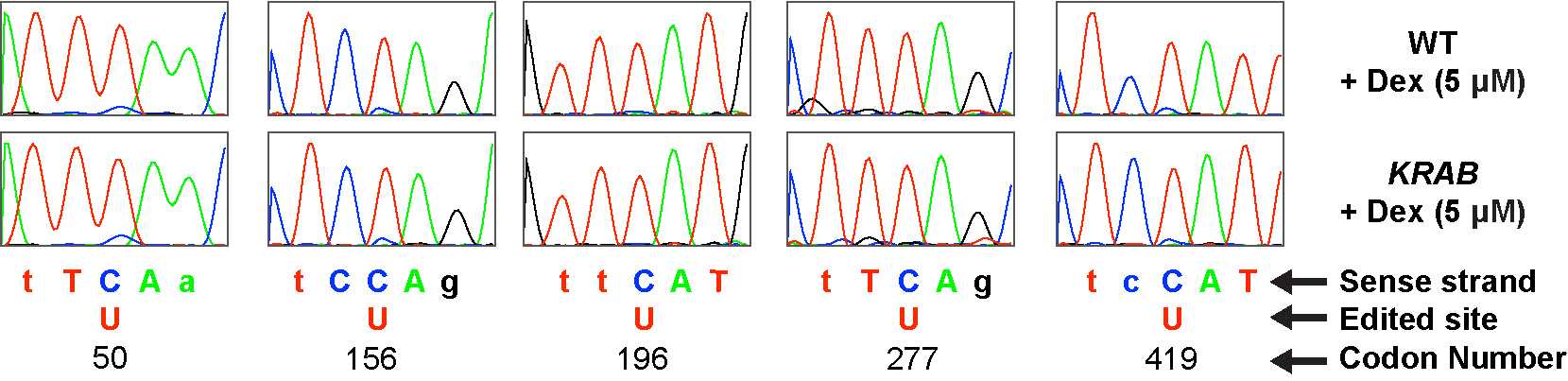

Supplement: Supplementary file 5 [file Image_4.tif]

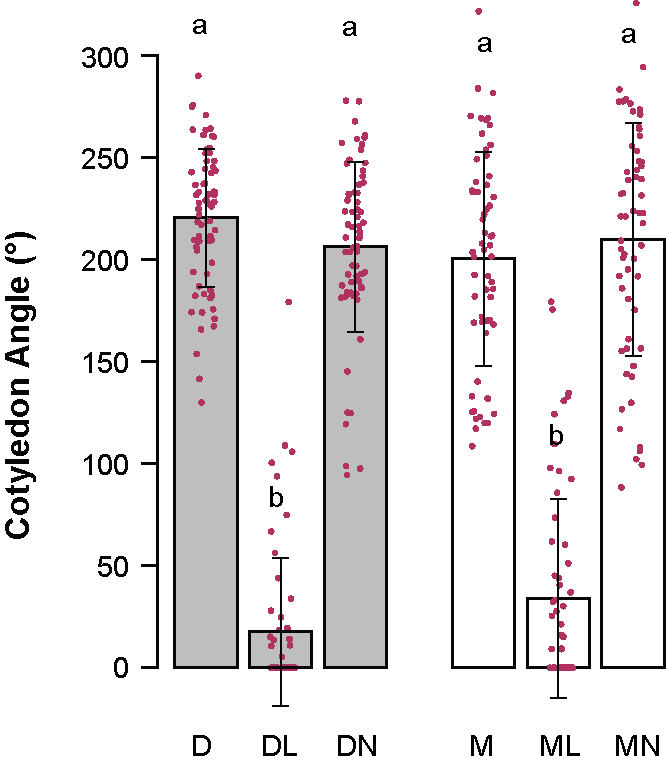

Supplement: Supplementary file 6 [file Image_5.tif]

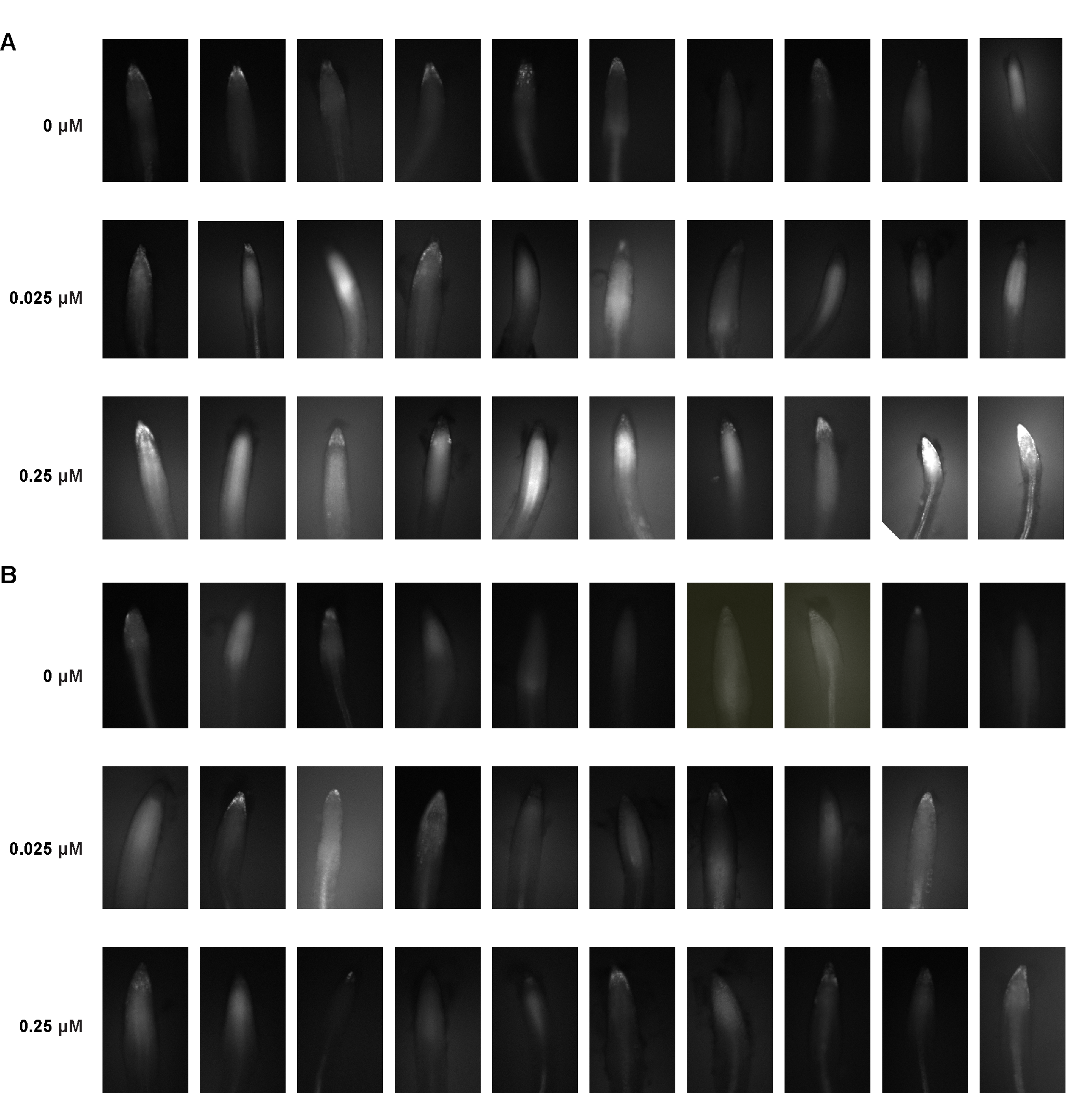

Supplement: Supplementary file 7 [file Image_6.tif]
